# Neural Control of Startle-Induced Locomotion by the Mushroom Bodies and Associated Neurons in *Drosophila*

**DOI:** 10.3389/fnsys.2018.00006

**Published:** 2018-03-28

**Authors:** Jun Sun, An Qi Xu, Julia Giraud, Haiko Poppinga, Thomas Riemensperger, André Fiala, Serge Birman

**Affiliations:** ^1^Genes Circuits Rhythms and Neuropathology, Brain Plasticity Unit, Centre National de la Recherche Scientifique, PSL Research University, ESPCI Paris, Paris, France; ^2^Department of Molecular Neurobiology of Behavior, Johann-Friedrich-Blumenbach-Institute for Zoology and Anthropology, University of Göttingen, Göttingen, Germany

**Keywords:** dopamine, mushroom bodies, startle-induced negative geotaxis, neural circuits, *Drosophila melanogaster*

## Abstract

Startle-induced locomotion is commonly used in *Drosophila* research to monitor locomotor reactivity and its progressive decline with age or under various neuropathological conditions. A widely used paradigm is startle-induced negative geotaxis (SING), in which flies entrapped in a narrow column react to a gentle mechanical shock by climbing rapidly upwards. Here we combined *in vivo* manipulation of neuronal activity and splitGFP reconstitution across cells to search for brain neurons and putative circuits that regulate this behavior. We show that the activity of specific clusters of dopaminergic neurons (DANs) afferent to the mushroom bodies (MBs) modulates SING, and that DAN-mediated SING regulation requires expression of the DA receptor Dop1R1/Dumb, but not Dop1R2/Damb, in intrinsic MB Kenyon cells (KCs). We confirmed our previous observation that activating the MB α'β', but not αβ, KCs decreased the SING response, and we identified further MB neurons implicated in SING control, including KCs of the γ lobe and two subtypes of MB output neurons (MBONs). We also observed that co-activating the αβ KCs antagonizes α'β' and γ KC-mediated SING modulation, suggesting the existence of subtle regulation mechanisms between the different MB lobes in locomotion control. Overall, this study contributes to an emerging picture of the brain circuits modulating locomotor reactivity in *Drosophila* that appear both to overlap and differ from those underlying associative learning and memory, sleep/wake state and stress-induced hyperactivity.

## Introduction

The identification of neural circuits that modulate innate or reflex behaviors is essential to better understand how the brain functions and adapts to a changing environment (LeBeau et al., 2005; Dickinson, 2006; Marder, 2012; Su and Wang, 2014). *Drosophila* is an advantageous organism for studying the neural basis of behavior using genetically-encoded probes that enable *in vivo* control of neuronal activity (White and Peabody, 2009; Griffith, 2012; Yoshihara and Ito, 2012; Kazama, 2015; Owald et al., 2015b; Riemensperger et al., 2016; Martín and Alcorta, 2017). In this organism, spontaneous locomotor activity and locomotor reactivity have been described as two separate behavioral systems that are regulated differently (Connolly, 1967; Meehan and Wilson, 1987; O'Dell and Burnet, 1988; Martin et al., 1999a). A sudden external stimulus (startle) usually triggers inhibition or arrest of spontaneous locomotion followed by an appropriate behavioral response, which may itself be a locomotor reaction. Startle-induced reactivity has long been used in *Drosophila* to monitor various behavioral performances, such as phototaxis (Benzer, 1967) or negative geotaxis (Miquel et al., 1972). A widely used paradigm relies on the fast climbing reaction initiated by a gentle mechanical shock of flies entrapped in a vial or a narrow column, an innate reflex called startle-induced negative geotaxis (SING). SING performance progressively declines with age (Ganetzky and Flanagan, 1978; Le Bourg and Lints, 1992; Grotewiel et al., 2005; White et al., 2010; Jones and Grotewiel, 2011; Vaccaro et al., 2017), in contrast to spontaneous locomotion that does not vary during the adult life and even increases in old flies (White et al., 2010). The SING reflex is also progressively altered in various mutant or under neuropathological conditions, as is the case in *Drosophila* models of Parkinson disease (Feany and Bender, 2000; Coulom and Birman, 2004; Chaudhuri et al., 2007; Riemensperger et al., 2013; Bou Dib et al., 2014). It is therefore of particular interest to identify precise neural components underlying the modulation of startle-induced locomotion, in *Drosophila* as in other species (Hale et al., 2016).

The mushroom body (MB) is a paired structure of the insect brain that has important behavioral functions, including the formation of olfactory memory (Heisenberg, 2003; Fiala, 2007; Davis, 2011; Kahsai and Zars, 2011; Waddell, 2013) and the control of sleep (Bushey and Cirelli, 2011; Tomita et al., 2017). The *Drosophila* MB is composed of intrinsic neurons known as Kenyon cells (KCs) and it is innervated by afferent modulatory neurons, in particular subsets of dopaminergic neurons (DANs), as well as efferent MB output neurons (MBONs) (Tanaka et al., 2008; Pech et al., 2013a; Aso et al., 2014a,b). The cell bodies of the KCs form a large cluster in the dorsal posterior brain; their dendritic branches make up the calyx and their axons bundle up in the peduncles. The KCs are named according to the lobes in which they send axonal projections: αβ, α'β', and γ (Lee et al., 1999; Tanaka et al., 2008). At the distal end of the peduncles, the axons of the αβ and α'β' KCs bifurcate dorsally and medially to form the vertical (α and α') and horizontal (β and β') lobes, while the γ KCs form only the γ horizontal lobes.

Around 60 years ago, experiments carried out on crickets provided the first evidence that the insect MB contains neurons inhibiting locomotion (Huber, 1960, 1967; Howse, 1975). In *Drosophila*, both the *mushroom body miniature* mutation or chemical ablation of the MB increased walking activity when measured over long time intervals, confirming that the MB normally suppresses locomotor behavior (Heisenberg et al., 1985; Martin et al., 1998; Helfrich-Förster et al., 2002), while similar experiments suggested that, by contrast, the MB stimulates initial stages of walking activity (Serway et al., 2009). Neuroanatomical defects in the MB lobes were observed in a set of mutants giving rise to changes in startle-induced locomotion behavior, but without a clear correlation between the two phenotypes (Yamamoto et al., 2008). Furthermore, we previously reported that SING is controlled by the activity of the α'β' KCs (Riemensperger et al., 2013). Determining the precise contributions of the various subtypes of MB neurons to startle-induced locomotion required, therefore, further investigations.

Dopamine (DA) is an important neurotransmitter that, in flies, was implicated in the modulation of diverse behaviors including appetitive or aversive learning (Schwaerzel et al., 2003; Riemensperger et al., 2005, 2011; Schroll et al., 2006; Claridge-Chang et al., 2009; Krashes et al., 2009; Aso et al., 2010; Waddell, 2010; Berry et al., 2012; Burke et al., 2012; Plaçais et al., 2012; Cohn et al., 2015; Musso et al., 2015; Aso and Rubin, 2016; Yamagata et al., 2016) and sleep-wake mechanisms (Van Swinderen and Andretic, 2011; Liu et al., 2012b; Ueno et al., 2012; Berry et al., 2015; Sitaraman et al., 2015b; Pimentel et al., 2016). It is also well established that DA prominently controls locomotor activity in *Drosophila* (Yellman et al., 1997; Bainton et al., 2000; Friggi-Grelin et al., 2003; Kume et al., 2005; Lima and Miesenböck, 2005; Wu et al., 2008; Lebestky et al., 2009; Kong et al., 2010; Riemensperger et al., 2011; Van Swinderen and Andretic, 2011) as it does in vertebrates (Beninger, 1983; Zhou and Palmiter, 1995; Giros et al., 1996; Blum et al., 2014). We have recently reported that the degeneration of DANs of either the protocerebral anterior medial (PAM) or protocerebral posterior lateral 1 (PPL1) clusters afferent to the MBs was associated with an accelerated decline of SING performance in aging flies (Riemensperger et al., 2013; Vaccaro et al., 2017). Further recent studies support a function for the PAM and PPL1 clusters in climbing or flight control (Bou Dib et al., 2014; Agrawal and Hasan, 2015; Pathak et al., 2015). However, the role of these and other DANs in SING modulation has not yet been precisely investigated.

Here we used activation or silencing of synaptic transmission in neuronal subsets targeted with selective drivers in order to identify the MB-associated neurons (KCs, DANs, and MBONs) that control startle-induced locomotion in *Drosophila*. Neuronal activation revealed that several classes of DANs projecting to the MBs have diverse roles in modulatory mechanisms. We show that DANs in the PPL1 cluster act as inhibitory neurons in the SING-modulating circuits, while the PAM cluster appears to contain both inhibitory and excitatory DAN subsets. We also confirm that MB α'β' KCs are implicated in SING control and demonstrate that γ KCs are involved in this modulation as well. Interestingly, we find that α'β' and γ neuron-mediated SING modulation is antagonized by co-activating the αβ KCs. Finally, we show that the MBONs M4/M6 and V2 are part of the network, suggesting that they convey SING modulatory information to downstream motor circuits. Overall, this work provides a first picture of the brain network and modulatory mechanisms controlling startle-induced locomotion in *Drosophila* that centrally involve a subset of MB-associated neurons.

## Materials and methods

### *Drosophila* culture and strains

Fly stocks were raised and crossed at 25°C on the standard corn meal/yeast/agar medium supplemented with methyl-4-hydroxybenzoate as a mold protector, under a 12 h/12 h light-dark cycle. The following effector lines were used: *UAS-mCD8::GFP, UAS-n-syb::GFP* (here named *UAS-msGFP*) (Riemensperger et al., 2013), *UAS-shi*^ts1^ (Kitamoto, 2001), *UAS-dTrpA1* (Hamada et al., 2008), *UAS-ChR2-XXL* (Dawydow et al., 2014), *LexAop-dTrpA1* (Burke et al., 2012), *UAS-Dumb-RNAi* (Bloomington *Drosophila* Stock Center, line 62193)*, UAS-Damb-RNAi* (Vienna *Drosophila* RNAi center, line v3391) (Cassar et al., 2015), *UAS-n-syb::spGFP*_*1-10*_, *LexAop-CD4::spGFP*_*11*_/*CyO* and *LexAop-n-syb::spGFP*_*1-10*_, *UAS-CD4::spGFP*_*11*_ (Bloomington *Drosophila* Stock Center, lines 64314 and 64315) (Macpherson et al., 2015). The driver lines used and their brain expression patterns are described in Table S1. Except for those that were generated in our laboratories, these lines were either obtained from the Bloomington *Drosophila* Stock Center or kindly provided by: Ronald L. Davis (*TH-LexA*, Berry et al., 2015), Thomas Preat and Pierre-Yves Plaçais (*4-59-Gal4, 238Y-Gal4, G0050-Gal4, NP2758-Gal4, R71D08-Gal4, NP2492-Gal4, R27G01-Gal4, R14C08-LexA*), Hiromu Tanimoto (*R58E02-Gal4*, Liu et al., 2012a) and Mark Wu (*TH-C1-Gal4, TH-C'-Gal4*, and *TH-D'-Gal4*) (Liu et al., 2012b).

### Locomotion assay coupled with genetic manipulation of neuronal activity

SING assays were generally carried out following thermogenetic inhibition or activation of neuronal activity. Seven- to ten-day-old flies expressing Shi^ts1^ or dTrpA1, respectively, or msGFP as a control, in neuronal subsets, were kept at 19°C overnight. The next day, groups of 10 flies of the same genotype were placed in a vertical column (25 cm long, 1.5 cm diameter) with a conic bottom end, and left for about 20 min at 19°C for habituation. Thermogenetic activation or silencing of neurons was performed by incubating each column for 10 min at 32°C, or at 23°C for control of a potential temperature effect. SING assays were carried out immediately afterwards at 23°C as previously described (Coulom and Birman, 2004; Riemensperger et al., 2013). Briefly, flies were suddenly startled by gently tapping them down. After 1 min, flies having reached the top of the column (above 22 cm) and flies remaining at the bottom end (below 4 cm) were separately counted. Three rounds of test were performed in a row per column. Results are the mean ± SEM of the scores obtained with ten groups of flies per genotype. The performance index (PI) is defined as $\frac{1}{2}$[(n_tot_ + n_top_ − n_bot_)/n_tot_], where n_tot_ is the total number of flies, and n_top_ and n_bot_ the number of flies at the top and at the bottom, respectively.

In some experiments, optogenetic photostimulation was performed instead on 7 to 10-day-old flies expresssing the channelrhodopsins *ChR2-XXL* (Dawydow et al., 2014) in neuronal subsets. In this case, flies were kept in constant darkness, and all manipulations before the SING assay were done under dimm red light. The transparent columns were introduced in a dark box and illuminated during locomotion testing with either blue-light diodes (peak wavelength 468 nm) from two sides (intensity range 6–11 × 10^3^ Lux), or red light as a control. Six rounds of tests were performed in a row per column, 3 under red light and 3 under blue light. Further details on the SING assay procedure under optogenetic photostimulation are provided in the legends to Figures S2A,B.

### Immunohistochemistry

Adult brains were dissected in ice-cold *Drosophila* Ringer's solution and processed for whole mount immunostaining as previously described (Riemensperger et al., 2011). The primary antibodies were mouse anti-GFP (ThermoFisher Scientific 33-2600, 1:500 for msGFP detection or Sigma-Aldrich G6539, 1:200 for reconstituted splitGFP (rsGFP) detection) and rabbit anti-TH (Novus Biologicals NB300-109, 1:1,000). The secondary antibodies were goat anti-mouse and anti-rabbit conjugated to Alexa fluor 488 or 555 (Invitrogen Molecular Probes, 1:1,000). The brains were mounted in ProLong Gold Antifade reagent (ThermoFisher Scientific). Images were acquired with a Nikon A1R confocal microscope and processed using the Fiji software (Schindelin et al., 2012).

For the quantification of Gal4 expression patterns in KC subpopulations, the brains of 5–7 day-old female flies expressing *mCD8::GFP* under the control of different Gal4 drivers were dissected in ice cold Ringer's solution, fixed for 2 h on ice in 4% paraformaldehyde and washed 3 × 20 min in phosphate-buffered saline + 0.6 % Triton X-100 (PBSTx). After a 2 h pre-incubation in PBSTx + 2% bovine serum albumin, brains were incubated overnight at 4°C in the same buffer with mouse monoclonal anti-Bruchpilot antibody (1:10, nc82, Developmental Studies Hybridoma Bank) to visualize synaptic neuropils. After 3 × 20 min washes in PBSTx, samples were incubated for 2 h with Cy3-conjugated anti-mouse secondary antibody (1:300, Jackson ImmunoResearch), then washed 3 × 20 min in PBSTx and additionally overnight in PBS. Brains were mounted in Vectashield (Vector Laboratories) and scanned using a Leica SP8 confocal laser scanning microscope equipped with hybrid detectors. Quantification of Gal4-expressing KC somata was conducted by monitoring GFP autofluorescence with the Fiji Cell Counter plugin across the focal planes.

### Split-GFP reconstitution

For the visualization of potential synaptic connectivity with the GRASP method (Feinberg et al., 2008; Gordon and Scott, 2009; Pech et al., 2013a; Macpherson et al., 2015), the *Drosophila* line *LexAop-n-syb::spGFP*_1−10_, *UAS-CD4::spGFP*_11_ was crossed to the recombined driver line *NP2492-Gal4*; *TH-LexA* (MBON-V2 and DANs), and the line *UAS-n-syb::spGFP*_1−10_, *LexAop-CD4::spGFP*_11_ was crossed to the recombined driver lines *R14C08-LexA*; *R58E02-Gal4* (MBON-M4/M6 and PAM DANs) and *NP2492-Gal4; R14C08-LexA* (MBON-V2 and MBON-M4/M6). 7–10 day-old female flies were collected for brain dissection followed by whole-mount brain immunostaining as described in the previous paragraph.

### Statistics

All statistical analyses were performed with the GraphPad Prism 6 software. Data from locomotor assays were analyzed using two-way ANOVA with Bonferroni's or Tukey's *post-hoc* tests for multiple comparisons. All data are presented as mean ± SEM. Significant values in all figures: ^*^p < 0.05; ^**^p < 0.01; ^***^p < 0.001.

## Results

### Activation of *TH-Gal4*-targeted DANs inhibits fly locomotor reactivity to startle

**Figure 1:**
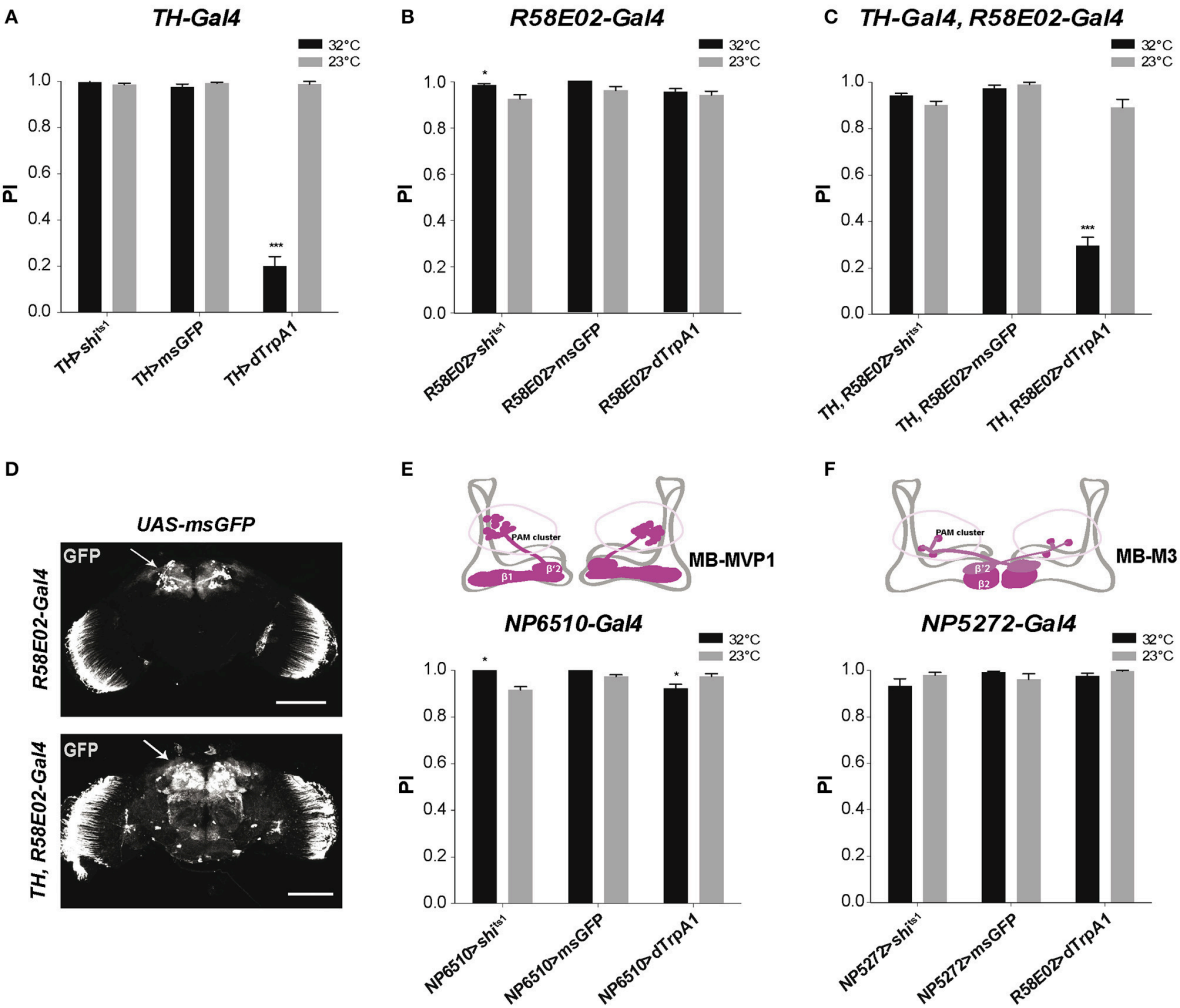
Differential modulation of *Drosophila* locomotor reactivity by brain DANs. **(A)** Thermoactivation of *TH-Gal4*-targeted neurons reduced SING performance of *TH*>*dTrpA1* flies at 32°C compared to the 23°C control. Expression of Shi^ts1^ or membrane-associated GFP (msGFP) had no consequence at 32°C, indicating that neither blocking neurotransmitter release in these neurons or temperature rise by itself alters SING. PI: performance index. **(B)** Thermoinhibition of PAM neurons targeted by *R58E02-Gal4* (*R58E02*>*shi*^ts1^ flies) at 32°C increased SING performance compared to the 23°C control, while thermoactivation of these neurons or temperature rise by itself (*R58E02*>*dTrpA1* and *R58E02*>*msGFP* flies, respectively) had no significant effects. **(C)** dTrpA1-mediated activation of all brain DANs using the *TH-Gal4, R58E02-Gal4* double driver decreased SING performance slighly less than *TH-Gal4* alone (shown in **A**) in parallel experiments (*p* < 0.1). Blocking with Shi^ts1^ synaptic output of all DANs at the restrictive temperature did not increase SING performance in contrast to the effect of *R58E02-Gal4* alone (shown in **B**). **(D)** Patterns of *R58E02-Gal4* and of the double driver *TH-Gal4, R58E02-Gal4* in the adult brain revealed by the expression of msGFP. The double driver labels all DANs including the PAM clusters (arrows). Scale bars represent 100 μm. **(E)** Thermogenetic inhibition or activation of *NP6510-Gal4*-targeted neurons increased and slightly decreased SING, respectively. This driver expresses in 15 PAM DANs including MB-MVP1 that project to the β1 and β'2 compartments in the horizontal lobes of the MBs (inset scheme) plus 3 non-DANs that target the fan-shaped body. **(F)** Inhibition or activation of PAM MB-M3 neurons targeted by *NP5272-Gal4* that project to the MBs in β2 and, more faintly, in β'2 (inset scheme) had no effect on SING performance. **(A–C,E,F)** Two-way ANOVA with Bonferroni's multiple comparisons tests (^*^p < 0.05; ^***^p < 0.001).

To determine the effect of DAN inhibition or activation on SING response, we first used *TH-Gal4*, a driver that expresses selectively in brain DANs, except in the PAM cluster where it only labels 12 DANs out of ~90 in total (Friggi-Grelin et al., 2003; Claridge-Chang et al., 2009; Mao and Davis, 2009; Aso et al., 2010; White et al., 2010; Pech et al., 2013a). We crossed *TH-Gal4* with *UAS-shi*^ts1^ flies to express the thermosensitive variant of *Drosophila* Dynamin Shi^ts1^ (Kitamoto, 2001) that blocks neurotransmitter release above 30°C (Kitamoto, 2001), in DANs of the progeny. After a 10-min incubation, these *TH*>*shi*^ts1^ flies showed no difference in SING performance between the permissive (23°C) and restrictive (32°C) temperatures, indicating that *TH-Gal4*-targeted DANs are not required for the execution of this locomotor response (Figure 1A). We checked that the *UAS-shi*^ts1^ transgene was active by expressing Shi^ts1^ in all neurons with *elav-Gal4*, which led to fly paralysis at the restrictive temperature (data not shown). Flies expressing a membrane-associated form of GFP (msGFP, described in section Materials and Methods) in *TH-Gal4* DANs neither showed any difference in SING performance between the two temperatures. This indicates that temperature by itself had no significant effect on the test (Figure 1A). In contrast, expressing the heat-inducible cation channel dTrpA1 (Hamada et al., 2008) in *TH-Gal4*-targeted DANs (*TH*>*dTrpA1* flies) led to altered SING performance after activation at 32°C, which was decreased to ~20% of the 23°C control value (Figure 1A). After 10 min of neuronal thermoactivation, *TH*>*dTrpA1* flies were in fact very active without any inhibition of their spontaneous locomotion (Movie S1). After the startle, most of these thermoactivated flies stayed at the bottom of the column and a few climbed up to the middle and stopped (Movie S2), while in the absence of neuronal thermoactivation, these same flies generally climbed to the top of the column quickly like wild-type flies (Movie S3). This indicates that DANs labeled by *TH-Gal4* inhibit the SING response i.e., locomotor reactivity, but not spontaneous locomotion, when they are stimulated.

In order to better characterize this behavioral modulation, we have monitored the SING performance of *TH*>*dTrpA1* flies after various times of incubation at 32°C (Figure S1A). We observed that 2 min were required for the temperature inside the column to reach above 30°C. Nervertheless, a decrease in SING performance could be observed after only 1 min of incubation, indicating that this modulation is actually rapid. SING performance continued to decrease until ~5 min of DAN thermoactivation, after what it remained stable at a low value (Figure S1A). Next, we checked whether DAN activation triggered during the climbing test could modulate as well SING behavior. We used for that optogenetic photostimulation in order to activate neurons instantly without the latency of thermoactivation, by expressing in DANs the channelrhodopsin ChR2-XXL (Dawydow et al., 2014; Riemensperger et al., 2016; Figures S2A,B). We first tested the efficiency of the system by expressing these optogenetic effectors in all GABAergic neurons with *Gad-Gal4*. As expected, blue light but not red light illumination after startle prevented *Gad*>*ChR2-XXL* flies from climbing (data not shown). Next we tested optogenetic stimulation of the DANs. We found that illuminating *TH*>*ChR2-XXL* flies with blue light, but not red light, during the test, i.e., within less than 1 min, was sufficient to reduce significantly their SING performance by ~22% (Figure S2C). These results indicate that DAN-mediated SING modulation is a fast and physiologically-relevant process.

### DANs in the PAM cluster are also involved in SING modulation

Because the *TH-Gal4* pattern excludes a large part of the PAM clusters, we used the *R58E02-Gal4* driver that labels ~80% of the PAM DANs (Liu et al., 2012a; Pech et al., 2013a) to investigate the role of this cluster in SING modulation. Again, no effect of temperature was detected in control *R58E02*>*msGFP* flies expressing msGFP in the PAM neurons (Figure 1B). Stimulating PAM DANs activity by dTrpA1 caused no inhibitory effect on fly locomotion, whereas blocking output from these neurons with Shi^ts1^ led to a small but statistically significant increase in SING performance at 32°C compared to 23°C (Figure 1B). This result suggests that the PAM clusters contain neurons that inhibit locomotor reactivity. These neurons appear spontaneously active during the test because their blockade by Shi^ts1^ increased SING while their stimulation by dTrpA1 did not lead to any effect. Indeed, it has recently been shown that some PAM DANs are spontaneously active (Yamagata et al., 2016).

We then constructed a double-driver strain containing both *TH-Gal4* and *R58E02-Gal4*. We checked that this double driver labeled all brain DANs, including the PAM clusters, by expressing msGFP and comparing to the pattern of the *R58E02-Gal4* strain (Figure 1D). Like with *TH-Gal4* alone, *TH, R58E02*>*shi*^ts1^ and *TH, R58E02*>*msGFP* flies showed similar SING performance at low and high temperatures. In contrast, *TH, R58E02*>*dTrpA1* flies showed at 32°C a climbing performance that was reduced to ~33% of the 23°C control value (Figure 1C), an effect that was slightly but significantly lower compared with the decrease observed in a parallel experiment with *TH-Gal4* alone (19.7 ± 4.5% vs. 33.1 ± 4.0% of the 23°C control for *TH*>dTrpA1 and *TH, R58E02*>*dTrpA1* flies, respectively). This result suggests that PAM neuron co-stimulation somewhat offsets the inhibition of locomotor reactivity induced by *TH-Gal4*. It therefore seems that the PAM clusters contain not only neurons that constitutively inhibit locomotor reactivity, but also neurons that, on the other hand, increase SING when stimulated. The PAM clusters are known, indeed, to include functionally heterogeneous subsets of DANs (Liu et al., 2012a; Waddell, 2013).

The driver *NP6510-Gal4* expresses in 15 PAM DANs that are not labeled by *TH-Gal4* and that project to the MB horizontal lobe β1 and β'2 compartments (Figure 1E; Aso et al., 2010; Riemensperger et al., 2013). We previously showed that the degeneration of these 15 DANs induced by mutant α-synuclein accumulation led to progressive SING defects that were as strong as those observed by expressing mutant α-synuclein in all neurons of the fly (Riemensperger et al., 2013). This suggested that *NP6510-Gal4* DANs could be involved in SING modulation. *NP6510*>*shi*^ts1^ flies showed indeed a slight increase in SING performance at the restrictive temperature, similar to the effect observed with *R58E02-Gal4*, whereas dTrpA1-induced thermostimulation of these neurons by contrast led to decreased SING response (Figure 1E). No such effects were observed with *NP5272-Gal4* that expresses in three PAM cells involved in aversive odor memory, the MB-M3 neurons, which innervate the tip of the MB horizontal lobes (β2 and β'2 compartments) and are labeled by *TH-Gal4* (Aso et al., 2010; Figure 1F). Neither did a *NP6510-Gal4, R58E02-Gal80* recombinant driver that expresses only in three *NP6510*-targeted non-DANs have any effect on SING (data not shown). Our results suggest, therefore, that the PAM neurons that inhibit SING correspond to the *NP6510*-targeted DANs or a subset of these cells.

### MB-afferent DANs of the PPL1 clusters inhibit the SING response

**Figure 2:**
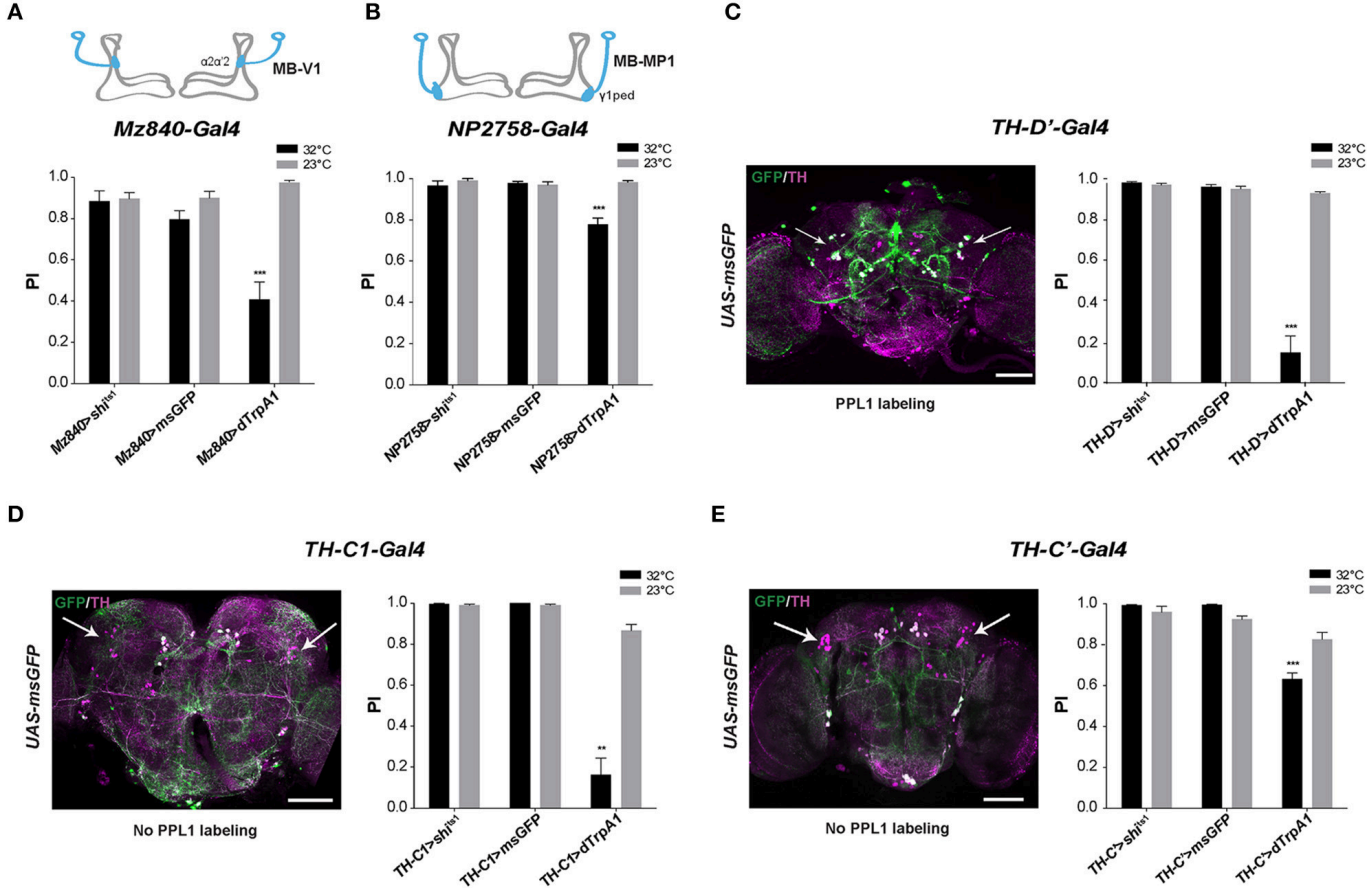
SING response decrease upon activation of MB-afferent PPL1 and other DANs. **(A)** Thermoactivation of the MB-V1 DANs in the PPL1 cluster with *Mz840-Gal4* (*Mz840*>*dTrpA1* flies) reduced locomotor reactivity, whereas blocking neurotransmitter release in these neurons (*Mz840*>*shi*^ts1^ flies) had no effect. MB-V1 targets the α2, α'2 compartments of the MB vertical lobes (inset scheme). **(B)** A decrease in SING performance was also observed with *NP2758-Gal4* that labels the PPL1 MB-MP1 neurons. MB-MP1 sends projections to the MB γ1 peduncle (γ1ped) (inset scheme). **(C)** Expression pattern of the *TH-D'-Gal4* driver is shown in Left. Nearly all PPL1 neurons are labeled (white arrows). SING was decreased after thermoactivation of *TH-D'-Gal4*-targeted neurons (Right). **(D)** *TH-C1-Gal4* does not label the PPL1 cluster (Left, arrows) whereas this driver expresses in the PPL2ab cluster and other DANs. Neuronal activation with *TH-C1-Gal4* markedly decreased SING performance (Right). **(E)** *TH-C'-Gal4* that does not label the PPL1 cluster as well (Left, arrows) and gave a lower but significant SING modulation (Right). Inhibition of the synaptic output using Shi^ts1^ had no effect with either *TH-C1-Gal4* or *TH-C' -Gal4*. Scale bars represent 100 μm. **(A–E)** Two-way ANOVA with Bonferroni's multiple comparisons tests (^**^p < 0.01; ^***^p < 0.001).

We recently reported that the progressive degeneration of DANs in the PPL1 clusters induced by a mutation of the circadian gene *Clock* severely accelerates age-related SING decline (Vaccaro et al., 2017). To identify whether PPL1 plays a direct role in SING modulation, we employed two drivers that label specific neurons in this cluster: *Mz840-Gal4* labeling the MB-V1 neuron that projects to MB dorsal lobes α2, α'2 compartments and *NP2758-Gal4* that expresses in the MB-MP1 neuron sending projection to the γ1 peduncle (Figures 2A,B; Aso et al., 2010, 2012). Whereas, the inhibition of the neurons targeted by each of these drivers had no effect on SING, their thermoactivation significantly decreased performance of the flies to around 41 and 78% of the 23°C control value for *Mz840-Gal4* and *NP2758-Gal4*, respectively (Figures 2A,B). We then used the driver *TH-D'-Gal4* (Liu et al., 2012b) that expresses strongly in the PPL1 cluster (Figure 2C). SING performance of *TH-D'*>*dTrpA1* flies at 32°C was markedly reduced to ~16% of the 23°C control (Figure 2C), an effect comparable to that of *TH-Gal4* itself (see Figure 1A). However, *TH-D'-Gal4* expresses in other DAN clusters than the PPL1 such as PPM2 and PPM3 (Liu et al., 2012b) that could contribute as well to SING modulation.

### DANs localized in other clusters are also implicated in SING regulation

To determine whether other DANs modulate the SING response, we selected two drivers, *TH-C1-Gal4* and *TH-C'-Gal4*, both of which do not express in the PPL1 (Liu et al., 2012b). We first verified that the PPL1 clusters were not labeled by these drivers (Figures 2D,E, left). The use of these drivers did not cause any effect on SING upon synaptic blockade with Shi^ts1^ but induced down-regulation of SING upon neuronal thermoactivation, which was strong with *TH-C1-Gal4* (18% of the 23°C control) (Figure 2D, right) and lower, but still significant, with *TH-C'-Gal4* (77% of the control) (Figure 2E, right). Both drivers express similarly in the protocerebral anterior medial (PAL), PPL2 and PPM2 DAN clusters, indicating that some of these clusters, and possibly the PPL2ab neurons that project to the MB calyx (Mao and Davis, 2009), could also be involved in SING modulation. Overall, our results suggest that several brain DAN subsets have the ability to hinder SING behavior when activated or inhibited, indicating that DA-mediated modulation of locomotor reactivity is an important and complex process in the insect brain.

### Activation of MB α'β' and γ neurons decreases SING performance

**Figure 3:**
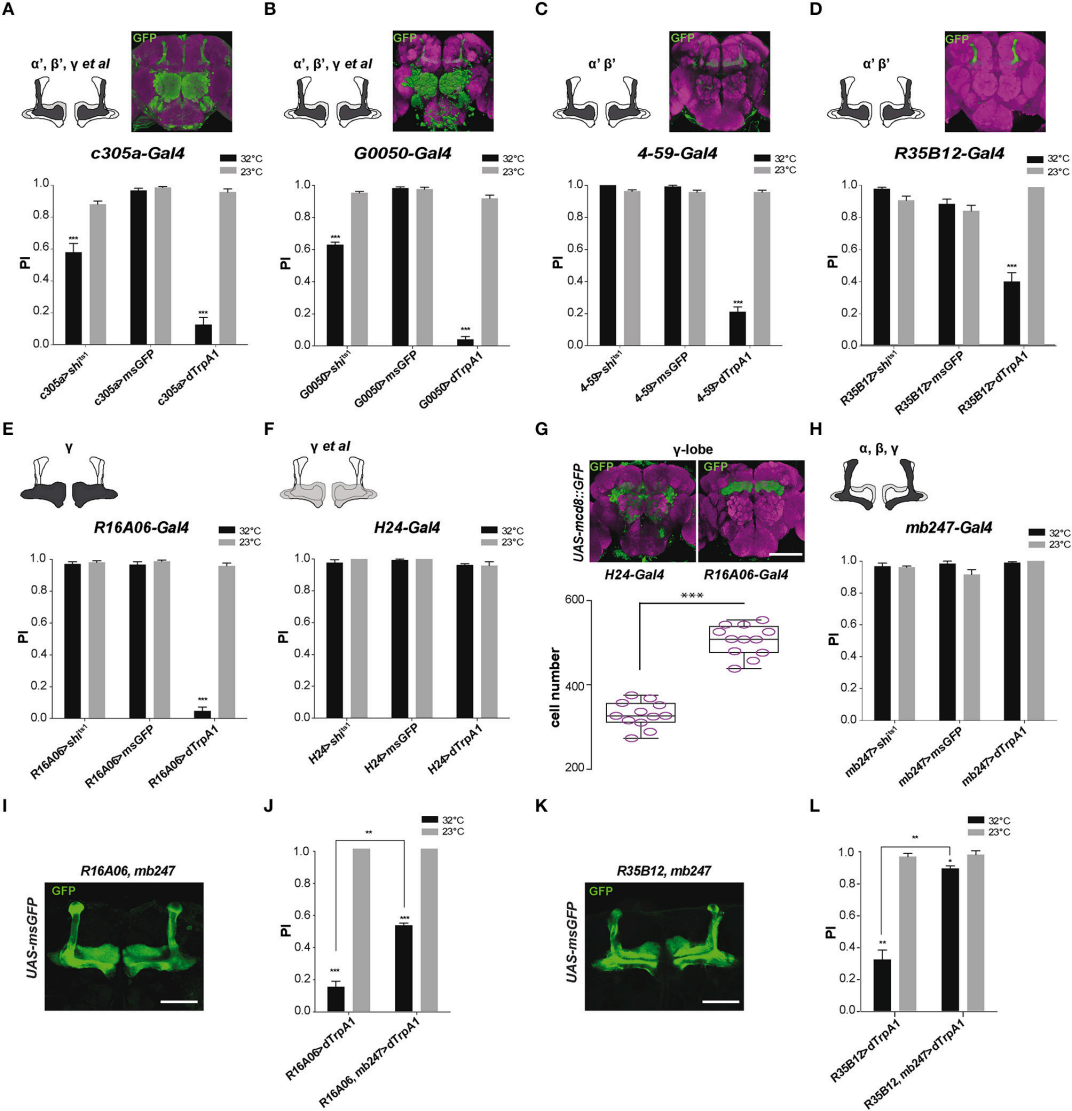
MB αβ neurons counteract SING modulation induced by α'β' and γ neuron activation. **(A–D)** Effect of drivers targeting α'β' lobe KCs. **(A,B)** Thermogenetic activation or synaptic inhibition of neurons labeled with *c305a-Gal4* or *G0050-Gal4* both decreased SING performance, with a stronger effect resulting from their activation. **(C,D)** With *4-59-Gal4* or *R35B12-Gal4*, SING was also reduced upon activation, but not upon block of synaptic output. Note that *c305a-Gal4* and *G0050-Gal4* labels other brain neuropils, whereas *4-59-Gal4* and *R35B12-Gal4* are very specific for the MB prime lobes. **(E,H)** Effect of drivers targeting γ lobe KCs. **(E)** Neuronal activation with the γ driver *R16A06-Gal4* strongly affected SING, while flies had normal response after inhibition of these neurons. **(F)** The use of another γ lobe driver, *H24-Gal4*, did not cause any effect on SING performance. **(G)** Analysis of expressions patterns in the brain indicates that *R16A06-Gal4* is very selective and expresses stronger than *H24-Gal4* in the γ lobe. Scoring the cells showed that *R16A06-Gal4* labels a larger number of γ KCs cells than *H24-Gal4*. **(H)** Neuronal activation or inhibition of MB αβ and γ lobes with *mb247-Gal4* did not modulate fly locomotor reactivity. **(I)** Expression pattern of the recombined double driver *R16A06-Gal4, mb247-Gal4* as revealed by msGFP expression. **(J)** Parallel experiment were performed to compare the effects on locomotor reactivity resulting from neuronal activation by *R16A06-Gal4* and the double driver *R16A06-Gal4, mb247-Gal4*. The SING decrease induced by *R16A06-Gal4, mb247-Gal4* in the first round of test was significantly mitigated compared to that induced by *R16A06-Gal4* alone. This suggests that γ lobe-induced SING modulation is inhibited by simultaneous αβ lobe activation. **(K)** Expression pattern of the recombined double driver *R35B12-Gal4, mb247-Gal4* as revealed by msGFP expression. **(L)** Parallel experiment was performed to compare the effects on locomotor reactivity resulting from neuronal activation by *R35B12-Gal4* and the double driver *R35B12-Gal4, mb247-Gal4*. Flies with neuronal activation in both αβγ and α'β' with *R35B12-Gal4, mb247-Gal4* showed normal SING performance compared to reduced performance in *R35B12*>*dTrpA1* flies. This suggests that activation of αβγ KCs blocked SING modulation induced by α'β' KCs. Scale bars represent 100 μm. **(A–F,H)** Two-way ANOVA with Bonferroni's multiple comparisons tests (^***^p < 0.001). **(G)** One-way ANOVA with Tukey's multiple comparisons test (^***^p < 0.001). **(J,L)** Two-way ANOVA with Tukey's multiple comparisons tests (^*^p < 0.05; ^**^p < 0.01; ^***^p < 0.001).

We previously reported that SING performance was decreased when synaptic activity in the MB prime (α'β') lobes, targeted with *c305a-Gal4*, was either thermogenetically inhibited or stimulated, and that the defect was stronger in the latter case (Riemensperger et al., 2013). We confirmed those results in the present work using either *c305a-Gal4* or *G0050-Gal4*: both drivers did induce SING inhibition at 32°C either with Shi^ts1^ or with dTrpA1 (Figures 3A,B). *c305a-Gal4* labels the entire MB α'β' lobes and the γ lobes faintly, as well as the antennal lobes, the central complex and other neuropils (Krashes et al., 2007; Pech et al., 2013b), while *G0050-Gal4* selectively labels the α'β' lobes in the MB, and also the ellipsoid body and brain glial cells (Lin et al., 2007; Chen et al., 2012). To ascertain the role of the α'β' lobes in SING modulation, we used two other drivers, *4-59-G*al4 and *R35B12-Gal4*, that restrictedly express in the MB prime lobes (Figures 3C,D, insets). Neuronal activation within *4-59*-*Gal4*- and *R35B12-Gal4*-labeled KCs decreased SING to around 21 and 54% of the 23°C control value (Figures 3C,D), compared to around 12.5% with *c305a-Gal4* and 4% with *G0050-Gal4* (Figures 3A,B). In contrast, Shi^ts1^ expression with *4*-*59-Gal4* and *R35B12-Gal4* did not cause any decrease in SING behavior at the restrictive temperature (Figures 3C,D). This suggests that the α'β' KCs are rather involved in SING inhibition than activation, and that another, still unidentified, targeted neuropile must be responsible for the Shi^ts1^-induced decrease observed with *c305a-Gal4* and *G0050-Gal4* (Figures 3A,B).

As mentioned, *c305a-Gal4* expresses in the α'β' lobes and in the γ lobes faintly. To further investigate the role of γ lobe KCs, we used the drivers *R16A06*-*Gal4* and *H24*-*Gal4* that target selectively γ neurons in the MB. Their expression patterns are shown in Figure 3G. We obtained discrepant results. Expressing *dTrpA1* with *R16A06-Gal4* nearly abolished fly locomotor reactivity at 32°C to around 4% of the 23°C control (Figure 3E), while the same experiment performed with *H24-Gal4* had no effect on SING (Figure 3F). Such a difference prompted us to analyze more precisely the expression patterns of these γ lobe drivers. First, *H24-Gal4* also labels the αβ lobes slightly in contrast to *R16A06-Gal4* that appears selective for the γ lobes. Second, by counting the labeled MB neurons using two-photon microscopy, we found that *R16A06-Gal4* expresses in around 500 γ lobe KCs per hemisphere while *H24-Gal4* labels around 300 γ neurons only (Figure 3G). It is quite possible that *H24-Gal4* does not express in a specific subset of γ KCs involved in SING control that would be in contrast targeted by *R16A06-Gal4*.

The γ lobe driver *R16A06-Gal4* had such a strong effect that we looked more closely at SING modulation in *R16A06*>*dTrpA1* flies. Kinetics studies showed that the inhibition was fast with this driver indeed, decreasing SING performance to ~10% of controls after only 3 min of thermoactivation (Figure S1B). Optogenetic photostimulation of *R16A06*>*ChR2-XXL* flies during the climbing test was also able to reduce efficiently SING performance by ~30% (Figure S2D). Remarkably, at the end of a 10-min thermoactivation period, *R16A06*>*dTrpA1* flies were not paralyzed but in contrast very active in the column (Movie S4). After being tapped down, they did not start climbing, possibly because the startle stopped spontaneous locomotion while thermoactivation of the γ lobe prevented their locomotor reactivity (Movie S5). These experiments confirmed that γ lobe activation has a stronger effect on SING than DAN activation.

### αβ Lobe co-activation antagonizes SING modulation by α'β' and γ KCs

In our previous work, we considered that the αβ lobe neurons were not involved in SING modulation, because no effect could be seen after synaptic blockade or activation with *mb247-Gal4* that strongly targets the αβ and γ KCs (Riemensperger et al., 2013). Again, the result with *mb247-Gal4* could be confirmed here (Figure 3H). Similarly, the use of an αβ-specific driver, *c708a-Gal4*, did not induce any effect on SING (data not shown). Neuronal thermoactivation with *H24-Gal4* did not show any difference compared to the control, while that of *R16A06-Gal4*-targeted neurons led to a strong SING decrease (Figures 3E–G). Remarkably, both *mb247-Gal4* and *H24-Gal4*, which induce no effect on SING, express both in the αβ and γ KCs, whereas *R16A06-Gal4* that induce strong effect on SING targets the γ KCs selectively. This led us to the hypothesis that co-activation of αβ neurons could potentially antagonize SING modulation caused by γ lobe activation.

To test this possibility, a recombined *R16A06-Gal4, mb247-Gal4* double driver line was constructed. The pattern of this driver, as characterized by msGFP expression, showed even and strong α, β and γ lobe labeling (Figure 3I). Expressing *dTrpA1* with *R16A06-Gal4* confirmed the decreased fly locomotor reactivity at 32°C (15% of the 23°C control, Figure 3J), while neuronal activation of KCs targeted by the *R16A06-Gal4, mb247-Gal4* double driver in a parallel experiment showed remarkably rescued SING response that rose up to 53% of the control in the first round of test (Figure 3J). The response of these flies then declined in the two subsequent tests, possibly related to a dominant effect of *R16A06-Gal4*-induced neuronal activation. This result indicates that co-activating the αβ lobes can at least transiently inhibit SING blockade induced by activation of the γ lobe intrinsic neurons.

We then checked if activation of the αβ KCs could similarly interfere with SING modulation induced by α'β' KC activation. A recombined *R35B12-Gal4, mb247-Gal4* double driver line was constructed that strongly expresses in the αβ, γ and α'β' KCs, i.e., in all the MB lobes (Figure 3K). Strikingly, the significant effect of α'β' neuron thermoactivation by *R35B12-Gal4* on SING modulation (reduction of the response to 33% of the 23°C control) was nearly abolished when the double-driver *R35B12-Gal4, mb247-Gal4* was used in a parallel experiment (reduction to 90.5% of the control only) (Figure 3L). Therefore, co-activation of the αβ and γ neurons blocked the inhibitory effect induced by α'β' neuron activation. Accordingly, we observed that thermoactivation or synaptic blockade with a driver that expresses specifically in all MB lobes, *VT30559-Gal4*, only had little effects on SING modulation (data not shown). Overall, these results indicate that activity of the αβ KCs potently counteracts by an unknown mechanism the behavioral modulation induced by the α'β' and γ KCs.

### Regulation of locomotor reactivity requires DA receptor signaling in the MB

**Figure 4:**
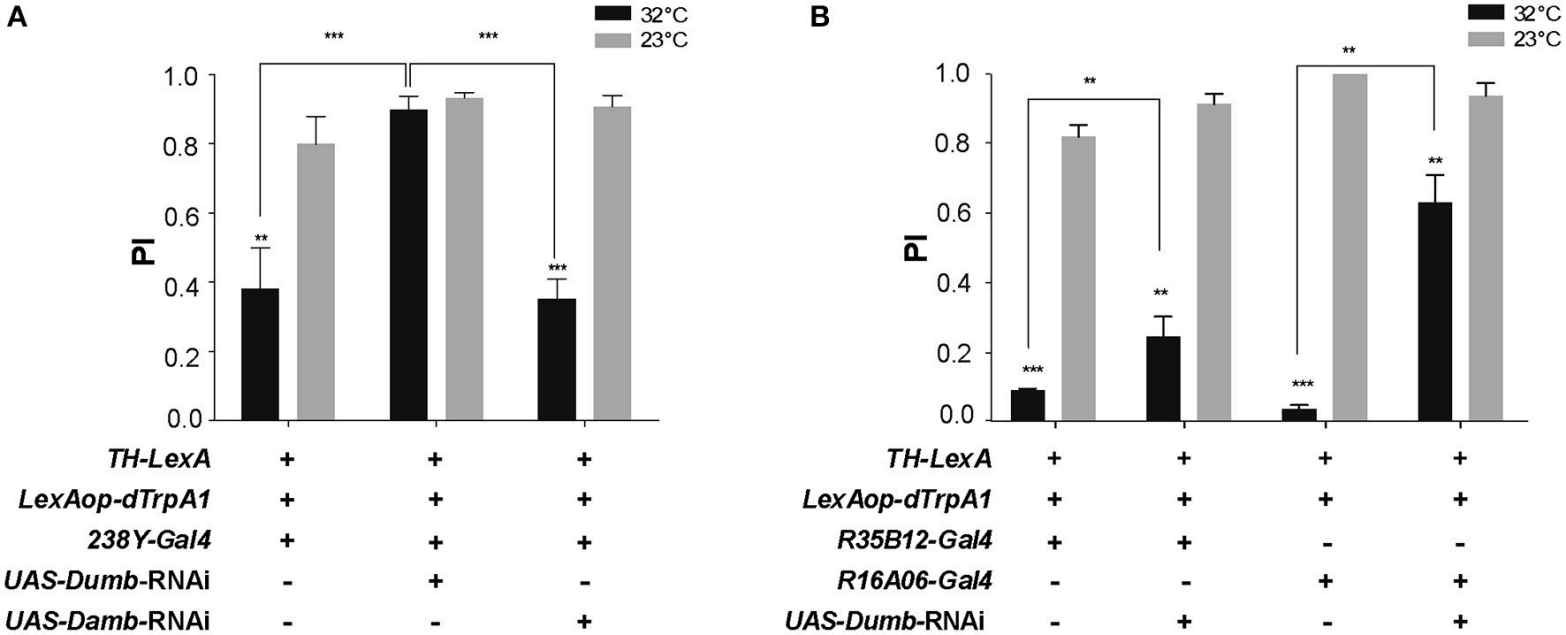
DA control of SING requires expression of the DA receptor Dop1R1/Dumb in the MB. **(A)** SING modulation was induced by DAN activation at 32°C in *TH-LexA*>*LexAop-dTRPA1* flies, but was prevented when Dumb expression was inhibited by RNAi in all MB KCs with *238Y-Gal4*. In contrast, RNAi inactivation of Dop1R2/Damb had no effect. **(B)** Similar experiments performed with the γ lobe driver *R16A06-Gal4* and the α'β' driver *R35B12*-*Gal4*. RNAi-mediated *Dumb* inactivation in both these KC subsets partially inhibited SING modulation induced by DAN thermoactivation. **(A,B)** Two-way ANOVA with Tukey's multiple comparisons tests (^**^p < 0.01; ^***^p < 0.001).

We next investigated whether down-regulation of DA receptor expression in the MB could prevent the decrease in SING caused by thermoactivation of DANs. Two DA receptors, D_1_-like Dumb/Dop1R1 and D_1/5_-like Damb/Dop1R2, are abundant in the MB lobes where they play key roles in olfactory memory (Kim et al., 2007; Seugnet et al., 2008; Selcho et al., 2009; Berry et al., 2012; Musso et al., 2015; Plaçais et al., 2017). Dumb has also been implicated in arousal and grooming (Andretic et al., 2008; Lebestky et al., 2009; Pitmon et al., 2016) and Damb in paraquat- and DA-induced neurotoxicity (Cassar et al., 2015). Taking advantage of the *LexA-LexAop* and *Gal4-UAS* expression systems, we expressed dTrpA1 in DANs using *LexAop-dTrpA1* and the *TH-LexA* driver, whose expression pattern is similar to that of *TH-Gal4* (Berry et al., 2015), while inactivating by targeted RNA interference (RNAi) the genes encoding Dumb or Damb in all MB lobes with the *238Y-Gal4* driver. As shown in Figure 4A, *TH-LexA*-controlled *dTrpA1* expression in the presence of *238Y-Gal4* alone induced a significant decrease in SING performance at 32°C (~48% of the 23°C control value). We observed that adding the *UAS-Dumb-RNAi* construct to allow *Dumb* inactivation in the MB fully restored SING performance to control level despite DAN thermoactivation (Figure 4A). In contrast, selective *Damb* inactivation had no such effect (Figure 4A). This experiment suggests that DA modulation of SING requires DA receptor expression in the MB KCs and that this regulation specifically depends on signaling through the Dumb receptor.

Next we investigated whether RNAi-mediated inactivation of Dumb expression in specific MB lobes could have a similar antagonistic effect on DA modulation of SING. We found that targeting *Dumb* RNAi selectively in the α'β' or γ lobes using *R35B12-Gal4* and *R16A06-Gal4*, respectively, in both cases significantly rescued the SING response, in spite of *TH-LexA*-mediated DAN activation (Figure 4B). This effect was most prominent with the strong and specific γ driver *R16A06-Gal4* (Figure 4B). This indicates a requirement for the DA receptor Dumb in the α'β' and γ lobes for DAN-mediated SING modulation.

### MBON-M4/M6 and MBON-V2 relay SING modulation

**Figure 5:**
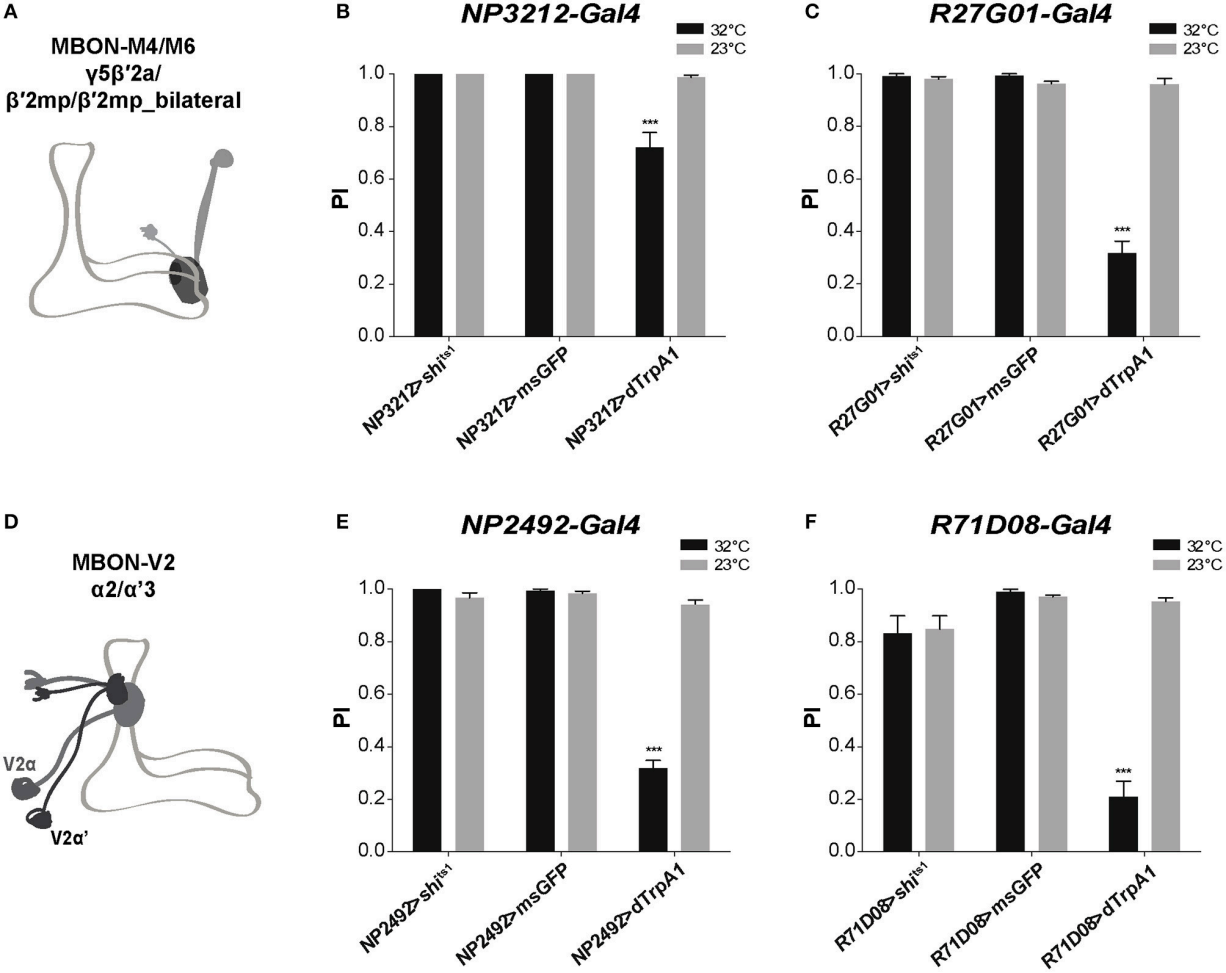
MB efferent neurons are part of the neuronal network for SING modulation. **(A)** Dendrites of the glutamatergic MBON-M4/M6 arborize on the tip of the MB horizontal lobes (γ5β′2a, β′2mp, β′2mp_bilateral compartments). **(B,C)** Thermoactivation with either *NP3212-Gal4 or R27G01-Gal4* that labels MBON-M4/M6 decreased the SING response, whereas neuronal thermoinhibition had no locomotor effect. **(D)** Dendrites of the cholinergic MBON-V2 arborize in the medial compartment of the MB vertical lobes (α2, α'3). **(E,F)** Thermoactivation with either *NP2492-Gal4* or *R71D08-Gal4* that labels MBON-V2 also markedly reduced SING performance, and again, inhibition of synaptic output had no effect. **(B,C,E,F)** Two-way ANOVA with Bonferroni's multiple comparisons tests (^***^p < 0.001).

We then attempted to identify specific MB-output neurons (MBONs) that could transfer MB modulatory information to downstream motor circuits. Since the intrinsic KCs in the MB α'β' and γ lobes appear to play a role in SING control, we studied the role of MBONs whose dendrites arborize on these lobes. The glutamatergic MBON-M4β, M4β' and M6 (also named MBON-β2β'2 and MBON-β'2mp for M4, and MBON-γ5β'2a for M6) arborize on the tip of the β, β', and γ lobes, respectively (Tanaka et al., 2008; Aso et al., 2014b; Owald et al., 2015a) (Figure 5A). These neurons are known to be involved in sleep regulation and the expression of appetitive and aversive memory performance (Aso et al., 2014b; Bouzaiane et al., 2015; Owald et al., 2015a; Sitaraman et al., 2015a). Using *NP3212-Gal4* and *R27G01-Gal4* that both target the MBON-M4 and M6 neurons (Tanaka et al., 2008; Bouzaiane et al., 2015), we observed that thermogenetic activation of these MB efferent neurons significantly reduced locomotor reactivity, while inhibiting their synaptic output with Shi^ts1^ had no effect (Figures 5B,C).

The cholinergic MBON-V2α and V2α' (also named MBON-α2sc and MBON-α'3, respectively) have their dendrites in the MB vertical lobes (α2, α'3) and are required for retrieval of aversive olfactory memory from the αβ lobe (Tanaka et al., 2008; Séjourné et al., 2011; Aso et al., 2014b; Bouzaiane et al., 2015; Figure 5D). Two specific drivers, *NP2492-Gal4* and *R71D08-Gal4* (Tanaka et al., 2008; Séjourné et al., 2011) were used to test whether V2 neurons are implicated in SING modulation. Activating these neurons with either of these drivers greatly reduced SING performance to around 33 and 21% of the 23°C control value, respectively, and again inhibition of synaptic output had no effect (Figures 5E,F). Finally, neither activation nor blocking of the MBON-V3 (alias MBON-α3) output, targeted by *G0239-Gal4*, had any effect on the SING response (data not shown), indicating that specific MBONs are involved in SING control. Hence, we propose that both MBON-M4/M6 and MBON-V2 participate in the transmission of MB regulatory information to the downstream SING reflex motor circuits.

### The ellipsoid body does not play a role in the modulation of startle-induced locomotion

**Figure 6:**
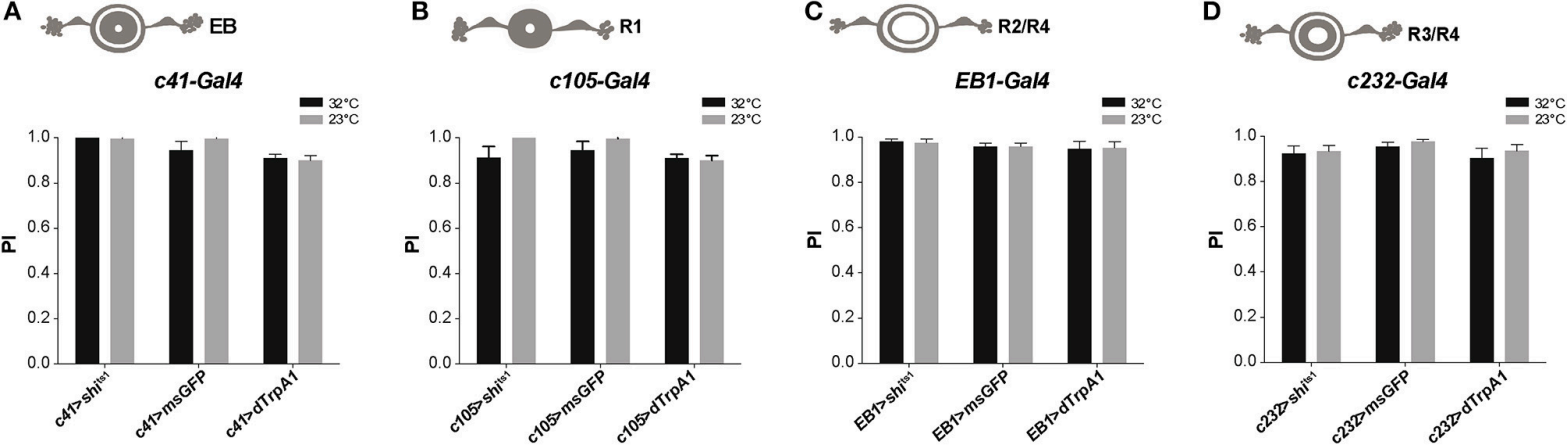
Activation or silencing of EB neurons has no effect on SING modulation. **(A–D)** Various drivers were used to inhibit synaptic output (with Shi^ts1^) or induce thermoactivation (with dTrpA1) in several classes of EB neurons: *c41-Gal4, c105-Gal4, EB1-Gal4* and *c232-Gal4*, that express in all **(A)**, R1 **(B)**, R2/R4 **(C)** and R3/R4 **(D)** EB neurons, respectively. No effect on SING performance could be observed in all cases.

The *Drosophila* ellipsoid body (EB) is a region of the central complex in the brain that controls locomotor patterns (Strauss and Heisenberg, 1993; Martin et al., 1999b, 2001; Strauss, 2002), as well as spatial orientation and visual pattern memories (Neuser et al., 2008; Pan et al., 2009). Subsets of DANs labeled by *TH-Gal4* heavily innervate the EB (Mao and Davis, 2009; White et al., 2010; Ueno et al., 2012; Riemensperger et al., 2013). Due to the complex structure of the EB, different driver lines have been used which express in various areas of the EB: *c41-Gal4* (all EB neurons), *c105-Gal4* (R1 neurons), *EB1-Gal4* (R2/R4d neurons), and *c232-Gal4* (R3/R4 neurons). Neuronal activation or synaptic inhibition with any of these drivers had no significant effect on the fly's locomotor reactivity, as tested by SING (Figure 6). This suggests that the EB is not involved in the neuronal circuits modulating startle-induced locomotion in *Drosophila*.

### Potential synaptic convergence between DANs and MBONs controlling SING

According to the MB neuronal architecture reported by Aso et al. (2014a), dendrites from the PAM DANs mainly reside in the crepine (CRE) and superior medial protocerebrum (SMP) brain regions, and slightly also in the superior intermediate protocerebrum (SIP) and superior lateral protocerebrum (SLP). The PPL1 DANs have a large part of their dendrites in the SMP, which is also where the MBON-M4/M6 and MBON-V2 send axonal projections. In order to detect zones of potential synaptic connections between the afferent and efferent MB neurons, we used the technique of splitGFP reconstitution (also named GFP reconstitution across synaptic partners, GRASP) coupled with the *LexA-LexAop* and *Gal4-UAS* systems (Feinberg et al., 2008; Gordon and Scott, 2009; Pech et al., 2013a; Macpherson et al., 2015).

**Figure 7:**
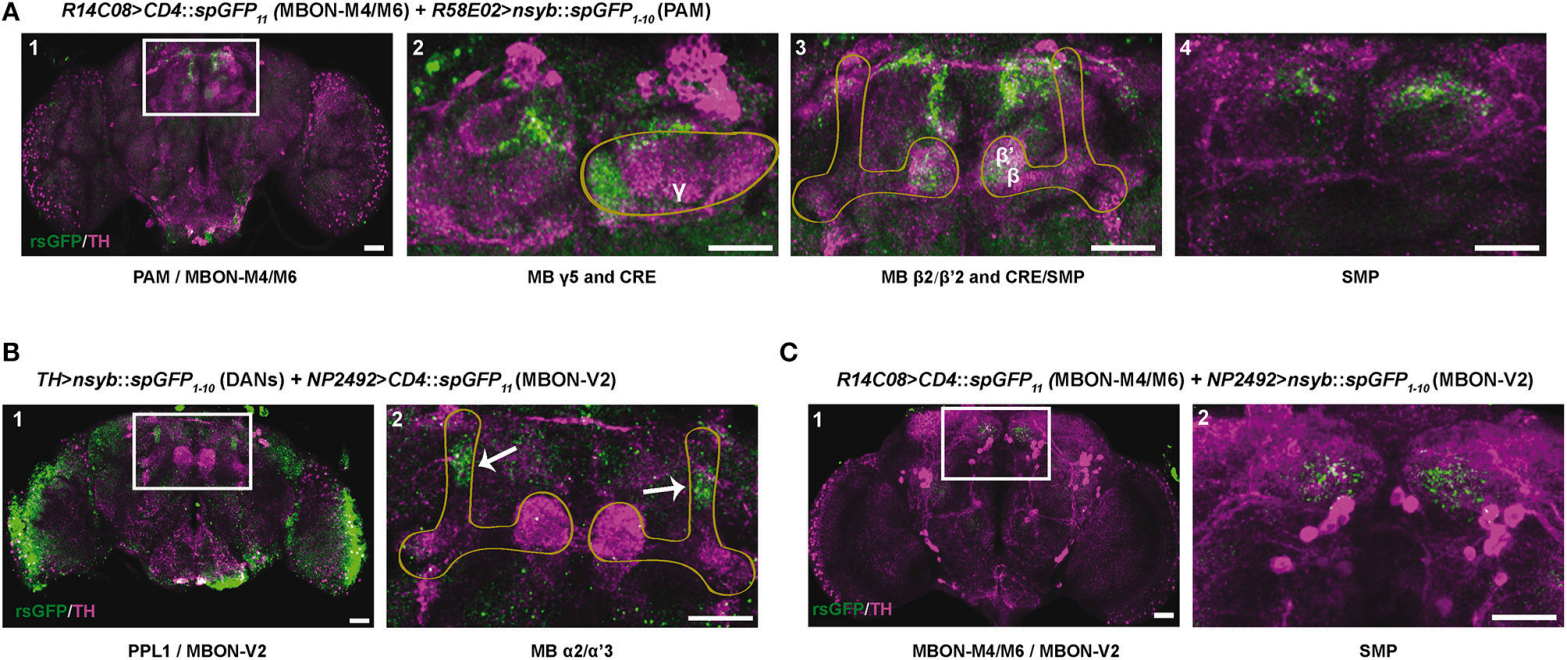
Identification of potential synaptic connections between SING modulatory neurons by splitGFP reconstitution. **(A)** Reconstituted splitGFP (rsGFP) signal between PAM DANs and glutamatergic MBON-M4/M6. n-syb::spGFP_1−10_ was expressed in PAM neurons with *R58E02-Gal4* and CD4::spGFP_11_ in MBON-M4/M6 with *R14C08-LexA*. rsGFP fluorescence localized at the tips of the MB horizontal lobes (γ5 and β2, β'2), as well as in the crepine (CRE) and superior medial protocerebrum (SMP) neuropiles where MBON-M4/M6 send their axonal projections. Panel A1 is a view of the whole brain. Panels 2–4 show different zoomed Z projections of the white box area in A1. **(B)** rsGFP signal between *TH-LexA*-targeted DANs and cholinergic MBON-V2 labeled with *NP2492-Gal4*. rsGFP fluorescence localized in the MB vertical lobes α2, α'3 compartments. Panel B2 is a magnification of the white box in B1. **(C)** rsGFP signal between MBON-M4/M6 and MBON-V2 labeled with *R14C08-LexA* and *NP2492-Gal4*, respectively. Localization of rsGFP fluorescence suggests the existence of axo-axonic synaptic connections between MBON-M4/M6 and MBON-V2 in the SMP. Panel C2 corresponds the white box in C1. Scale bars represent 30 μm.

The PAM DAN projections mainly tile the MB horizontal lobes where the MBON-M4/M6 dendrites arborize (Pech et al., 2013b; Riemensperger et al., 2013; Aso et al., 2014a). Results of splitGFP experiments indicated a potential synaptic convergence between these two groups of neurons in the tips of the MB horizontal lobes (γ5, β2, and β'2 compartments) (Figure 7A1–3) and also in the CRE and SMP neuropiles (Figure 7A2–4). This suggests, in agreement with a previous report (Owald et al., 2015a), that the zones of convergence between PAM and M4/M6 neurons not only localize in the MB horizontal lobes but also in the superior protocerebrum where the M4/M6 neurons appear to project onto the PAM DAN dendrites.

MBON-V2 arborizes on the MB vertical lobes (Tanaka et al., 2008; Séjourné et al., 2011; Aso et al., 2014b). Reconstituted split GFP (rsGFP) signals between MBON-V2 and DANs targeted by *TH-LexA* could be detected in the MB α and α' medial compartments, where the PPL1 MB-V1 neurons send projections (Aso et al., 2010, 2014b), indicating a close proximity between these neurons (Figure 7B1,2). A strong rsGFP signal was only observed when the presynaptic marker nsyb::spGFP_1−10_ was driven with *TH-LexA* and CD4::spGFP_11_ by the MBON-V2 driver *NP2492-Gal4* (Figures 7B1,2) and not the opposite (not shown), suggesting that DANs project to the MBON-V2 in the MB vertical lobe compartments. The occurrence of DAN>MBON synapses in the MB has recently been demonstrated in a comprehensive electron microscopy study (Takemura et al., 2017). Furthermore, rsGFP signals were visible between MBON-V2 and MBON-M4/M6 in the SMP region, which suggests that these MBONs may form axo-axonic reciprocal synapses (Figures 7C1,2). It seems that MBON-V2 could be presynaptic and MBON-M4/M6 postsynaptic in these contacts because a rsGFP signal in the SMP was only observed when the V2 driver *NP2492-Gal4* expressed the presynaptic marker nsyb::spGFP_1−10_ and the M4/M6 driver CD4::spGFP_11_ (Figures 7C1,2) and not the opposite (not shown). Therefore, there might be feedback signals from the MBON-V2 to MBON-M4/M6 and DANs that could optimize SING modulation, possibly in relation to learning and memory processes, and thus coordinate locomotor behavior with the environment.

## Discussion

In this study, we have identified MB afferent, intrinsic and efferent neurons that underlie modulation of startle-induced locomotion in the *Drosophila* brain. Using *in vivo* activation or silencing of synaptic transmission in neuronal subsets, we showed that specific compartments of the MBs are central to this modulation. Implicated neurons include α'β' and γ KCs, subsets of PAM and PPL1 DANs, and the MBONs V2 and M4/M6. We have also characterized some of the potential synaptic connections between these elements using splitGFP reconstitution across cells. Although the picture is not complete, these results led us to propose a first scheme of the neuronal circuits underlying the control of locomotor reactivity in an insect brain.

### DANs show diverse functions in the control of locomotor reactivity

We previously reported that the degeneration of DANs afferent to the MBs in the PAM and PPL1 clusters is associated with accelerated decline of SING performance in aging flies (Riemensperger et al., 2013; Vaccaro et al., 2017). Here we have specifically addressed the role of these and other DANs in SING modulation. Our initial observation was that thermoactivation of *TH-Gal4*-targeted DANs consistently led to decreased locomotor reactivity, while silencing synaptic output from these neurons had no effect. This result was verified by rapid optogenetic photostimulation, indicating that indeed DAN activation affects locomotor reactivity during the execution of the behavior. In contrast, blocking selectively synaptic output of the PAM DANs neurons resulted in a slight increase in SING performance, suggesting that a subset of spontaneously active neurons in the PAM inhibits SING. It should be noted, however, that this effect appeared small probably in part because SING performance was already very high for the control flies in our assay condition. This issue may have prevented us from detecting other modulatory neurons in the course of this study. Interestingly, our data suggest that those PAM neurons that inhibit SING are targeted by *NP6510-Gal4*, a driver that expresses in 15 PAM DANs that project to the MB β1 and β'2 compartments. The degeneration of these neurons also appears to be largely responsible for α-synuclein-induced decline in SING performance in a Parkinson disease model (Riemensperger et al., 2013). Moreover, we provided one observation in this study, using DAN co-activation with *TH-Gal4* and *R58E02-Gal4*, suggesting that other subsets of the PAM cluster may modulate locomotor reactivity with opposite effects, i.e., increase SING when they are stimulated.

Our study further indicated that thermoactivation of two DANs of the PPL1 cluster, either MB-MP1 that projects to the γ1 peduncle in the MB horizontal lobes or MB-V1 that projects to the α2 and α'2 compartments of the MB vertical lobes, was sufficient to significantly decrease SING performance. This suggests that the MB-afferent DANs of the PPL1 cluster are also implicated in SING modulation. Other DAN subsets could play a role and are still to be identified. However, inactivation of a DA receptor, Dop1R1/Dumb, in MB KCs precluded DAN-mediated SING modulation, strongly suggesting that DANs afferent to the MBs plays a prominent role in the neuronal network controlling fly's locomotor reactivity. In contrast, inactivating Dop1R2/Damb in KCs did not show any effect on DA-induced SING control.

Therefore, these results suggest that DA input to the MBs can inhibit or increase the reflexive locomotor response to a mechanical startle, allowing the animal to react to an instant, sudden stimulus. In accordance with this interpretation, previous reports have shown that the MB is not only a site for associative olfactory learning, but that it can also regulate innate behaviors (Hige et al., 2015; Lewis et al., 2015; Owald et al., 2015a). By combining synaptic imaging and electrophysiology, Cohn et al. (2015) have demonstrated that dopaminergic inputs to the MB intrinsic KCs play a central role in this function by exquisitely modulating the synapses that control MB output activity, thereby enabling the activation of different behavioral circuits according to contextual cues.

### Interactions between MB compartments contribute to SING modulation

We previously reported a decrease in SING performance when KCs in the α'β' lobes, but not in the αβ and γ lobes, were thermogenetically stimulated or their synaptic output silenced (Riemensperger et al., 2013). Here, using a set of specific drivers, we have more precisely studied the contribution of the various MB lobes in the modulation of this innate reflex. We confirmed that the α'β' KCs down-regulate SING when they are activated but not when their output is inhibited. Other unidentified neurons, which are targeted by the rather non-selective *c305a-Gal4* and *G0050-Gal4* drivers, trigger a decrease in SING performance when they are inhibited by Shi^ts1^, and are therefore potential SING-activating neurons. We further found that the MB γ lobes contain KCs that strongly inhibit SING when activated, both by thermogenetic and optogenetic stimulation, as shown with the γ-lobe specific driver *R16A06-Gal4*. However, thermoactivation of γ neurons with other drivers, like *mb247-Gal4*, which express both in the αβ and γ lobe, did not decrease SING (Riemensperger et al., 2013 and this study). This could result from an inhibitory effect of αβ neuron activation on SING modulation by γ neurons. To test this hypothesis, we have generated a double-driver by recombining *mb247-Gal4* with *R16A06-Gal4*. Because both drivers express in the γ lobes, one would expect a stronger effect on SING modulation after thermoactivation with the double-driver than with *R16A06-Gal4* alone. We observed strikingly the opposite, i.e., that SING was decreased to a less extent with the double-driver than with *R16A06-Gal4* alone. Activation of *mb247-Gal4* αβ neurons therefore likely counterbalanced the effect of γ neuron activation with *R16A06-Gal4* on SING modulation. A similar and even more obvious results was obtained when *mb247-Gal4* was recombined with the α'β' driver *R35B12-Gal4*: co-activation of the neurons targeted by these two drivers prevented the strong SING modulation normally induced by *R35B12-Gal4* alone. These results suggest the existence of an inter-compartmental communication process for locomotor reactivity control in the *Drosophila* MB. Comparably, it was recently suggested, in the case of memory retrieval, that MB output channels are ultimately pooled such that blockade (or activation) of all the outputs from a given population of KCs may have no apparent effect on odor-driven behavior, while such behavior can be changed by blocking a single output (Owald et al., 2015a). Such a transfer of information could occur, as was previously reported, through connections involving the MBONs within the lobes or outside the MB (Aso et al., 2014b; Owald et al., 2015a).

### Role of specific MBONs in innate reflex suppression

Finally, the activation of two sets of MB efferent neurons, cholinergic MBON-V2 and glutamatergic MBON-M4/M6, consistently decreased SING performance of the flies. In contrast, silencing these neurons had no effect on locomotor behavior, as was previously observed (Aso et al., 2014b). The dendrites of these MBONs arborize in the medial part of the vertical lobes (α2, α'3) and the tips of the horizontal lobes (β'2 and γ5), respectively, as a further evidence that the prime and γ lobes, and DANs efferent to these compartments, are involved in SING modulation. We also show results from GRASP observations suggesting that the PAM DANs lay very close or make potential synaptic connections with the MBON-M4/M6 neurons in their MB compartments, as well as the M4/M6 with the PAM in the SMP, in agreement with recent evidence from other laboratories (Lewis et al., 2015; Owald et al., 2015a; Takemura et al., 2017). Our results also provide evidence that the PPL1 DANs and MBON-V2 contact each other in the vertical lobes and that axo-axonic synaptic contacts may occur between the MBON-V2 and M4/M6 neurons in their common projection region in the SMP.

These MBONs are known to be involved in opposite ways in olfactory memory: DAN-induced synaptic repression of cholinergic or glutamatergic MBONs would result in the expression of aversive or attractive memory, respectively (Aso et al., 2014b). Here we find, in contrast, that the activation of these two sets of MBONs had similar depressing effects on SING behavior. Interestingly, it has been recently reported that the glutamatergic MBONs and PAM neurons that project to the MB β'2 compartment are also required for modulation of another innate reflex, CO_2_ avoidance (Lewis et al., 2015). CO_2_ exposure, like mechanical startle, represents a potential danger for the flies, thus triggering an avoidance behavior that can be suppressed by silencing these MBONs in specific environmental conditions. However, it is the activation of glutamatergic MBONs that inhibits SING. This apparent discrepancy might be explained if the downstream circuits were different for these two escape behaviors (CO_2_ avoidance and fast climbing). Overall, our results further support the hypothesis of a primary role of the MB as a higher brain center for adapting innate sensory-driven reflex to a specific behavioral context (Cohn et al., 2015; Lewis et al., 2015).

### Different neuronal circuits control locomotor reactivity, sleep/wake state and hyperactivity

**Figure 8:**
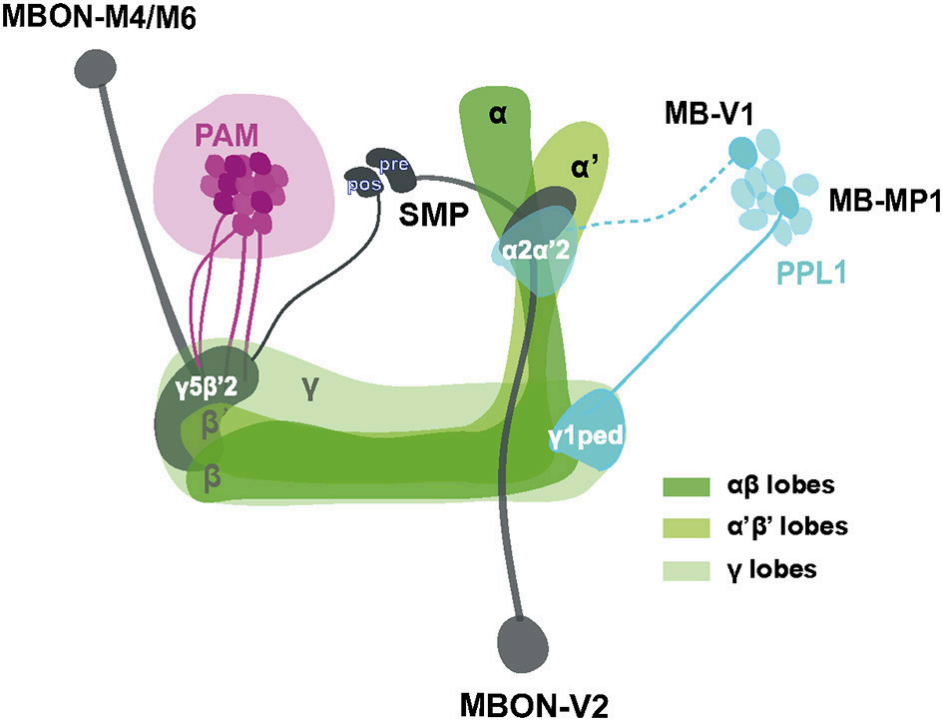
Schematic representation of MB-associated neural components modulating startle-induced locomotion. DA signals for SING modulation originate from PAM neuron subsets and neurons inside the PPL1 cluster (MB-MP1 and MB-V1) that project to the MB lobes. Axon of MB-V1 is shown as a dashed line because a driver specific for this neuron could not be tested in this study. The α'β' and γ KCs appear to be the main information integration center in this network, while their effect on SING modulation is opposed by the activity of αβ lobe KCs. Processed SING modulation signals are then transferred by two subtypes of MB efferent neurons, MBON-V2 and M4/M6, to the downstream SING reflex motor circuits. These two MBON subtypes have their axons converging together in the SMP where they may form axo-axonic synaptic connections, in which MBON-V2 would be presynaptic to MBON-M4/M6. The SMP also contains dendrites of the PAM and PPL1 DANs, thereby potentially forming instructive feedback loops on DA-mediated SING modulation. Most neurons identified here downregulated SING performance when they were activated, except for a subset of the PAM clusters that appeared constitutively inhibitory (represented as darker neurons in the figure) and the αβ lobe KCs that seem to antagonize SING modulation by other MB neurons. The different MB lobes are shown in various shades of green as indicated. The PAM DANs, PPL1 DANs and MBONs are drawn in magenta, light blue and dark gray, respectively. PAM: protocerebral anterior medial; PPL1: protocerebral posterior lateral; MBON: mushroom body output neuron; SMP superior medial protocerebrum; ped: peduncle; pre: presynaptic; pos: postsynaptic.

Even though the model remains to be confirmed and elaborated, a proposed scheme summarizing our current working hypothesis of the neural components underlying SING control is presented in Figure 8. Sensory information from mechanical stimulation triggers an innate climbing reflex (negative geotaxis) that can be regulated by signals transmitted from MB-afferent DANs (in the PAM and PPL1 cluster) to select KCs and two sets of MBONs (V2 and M4/M6) in specific MB compartments. Processing of this information could occur through synergistic or antagonistic interactions between the MB compartments and, finally, the MBON neurons would convey the resulting modulatory signal to downstream motor circuits controlling the climbing reflex. We observed that the axonal projections of these MBONs make synaptic contacts with each other and converge together to the SMP where the dendrites of DANs lie (Aso et al., 2014a), suggesting that they might form feedback loops to control DA signaling in the circuits.

SING performance can be affected by a collection of factors including the arousal threshold of the fly, the ability to sense gravity and also climbing ability. “Arousal” is defined as a state characterized by increased motor activity, sensitivity to sensory stimuli, and certain patterns of brain activity (Coull, 1998; Pfaff and Banavar, 2007). A distinction can be made between endogenous arousal (i.e., wakefulness as opposed to sleep) and exogenous arousal (i.e., behavioral responsiveness) (Van Swinderen and Andretic, 2011). In *Drosophila*, DA level and signaling control all known forms of arousal (Friggi-Grelin et al., 2003; Birman, 2005; Kume et al., 2005; Lebestky et al., 2009; Van Swinderen and Andretic, 2011; Kumar et al., 2012; Liu et al., 2012b; Ueno et al., 2012; Nall et al., 2016). Because the MB plays an important role in sleep regulation (Sitaraman et al., 2015a; Artiushin and Sehgal, 2017; Tomita et al., 2017), sleep- or wake-promoting networks might indeed in part interact or overlap with those controlling locomotor reactivity. However, we observed that thermoactivation with various drivers had in a number of cases opposite effects on sleep/wake state and SING. First, neuronal thermoactivation with *TH-Gal4* suppresses sleep (Liu et al., 2012b) but decreases the SING response. Second, extensive thermogenetic activation screen revealed that α′β′ and γm KCs are wake-promoting and γd KCs are sleep-promoting (Sitaraman et al., 2015a). In our experiments, neuronal activation of α′β′ or γ KCs both led to strongly decreased locomotor reactivity. Third, stimulating MBON-M4 and M6, which are wake-promoting (Sitaraman et al., 2015a), decreased SING performance.

Another brain structure, the EB, plays important roles in the control of locomotor patterns (Strauss, 2002) and is also sleep-promoting (Liu et al., 2016). Furthermore, the EB is involved in the dopaminergic control of stress- or ethanol-induced hyperactivity (Lebestky et al., 2009; Kong et al., 2010), which can be considered as forms of exogenously-generated arousal. We used several drivers labeling diverse EB neuronal layers and found no noticeable effects of thermoactivation of these neurons on the SING response. We conclude that the circuits responsible for SING modulation, although they apparently share some similarities, are globally different from those controlling sleep/wake state and environmentally-induced hyperactivity.

Overall, this work identified elements of the neuronal networks controlling startle-induced locomotion in *Drosophila* and confirmed the central role of the MBs in this important function. Future studies are required to complete this scheme and explore the intriguing interactions between the different MB compartments in SING neuromodulation.

## Ethics statement

Experiments on *Drosophila* are not subject to the approval of ethics committee. All experiments were nevertheless performed in accordance with ethic procedures and by minimizing the number of animals required for data gathering.

## Author contributions

JS, TR, AF, and SB: Conceived and designed the experiments; JS, AX, JG, HP, and TR: Performed the experiments; JS, AX, JG, HP, TR, AF, and SB: Analyzed data; JS, AX, TR, AF, and SB: Wrote the paper; SB: Designed and supervised the study.

### Conflict of interest statement

The authors declare that the research was conducted in the absence of any commercial or financial relationships that could be construed as a potential conflict of interest.

## Abbreviations

CRE: crepine
DA: dopamine
DAN: dopaminergic neuron
Dop1R1: Dopamine 1-like receptor 1
Dop1R2: Dopamine 1-like receptor 2
EB: ellipsoid body
dFSB: dorsal fan-shaped body
GFP: green fluorescent protein
GRASP: GFP reconstitution across synaptic partners
KC: Kenyon cell
MB: mushroom body
MBON: MB output neuron
msGFP, mCD8::GFP: n-syb::GFP
PAL: protocerebral anterior lateral
PAM: protocerebral anterior medial
PI: performance index
PPL: protocerebral posterior lateral
PPM: protocerebral posterior medial
RNAi: RNA interference
rsGFP: reconstituted splitGFP
SING: startle-induced negative geotaxis
SIP: superior intermediate protocerebrum
SLP: superior lateral protocerebrum
SMP: superior medial protocerebrum
TH: tyrosine hydroxylase.
