# Supplementary material for: Neural Control of Startle-Induced Locomotion by the Mushroom Bodies and Associated Neurons in Drosophila
Source: Front Syst Neurosci. 2018 Mar 28;12:6. doi: 10.3389/fnsys.2018.00006 (PMC5882849; doi:10.3389/fnsys.2018.00006)
Supplement: Supplementary file 6 [file DataSheet1.docx]

**Neural control of startle-induced locomotion by the mushroom bodies and associated neurons in *Drosophila***

**Jun Sun, An Qi Xu, Julia Giraud, Haiko Poppinga, Thomas Riemensperger, André Fiala and**

**Serge Birman^*^**

**SUPPLEMENTARY MATERIAL**

**- Figures S1 and S2**

**- Movie Captions**

**- Table S1**

**- Supplementary References**

**Supplementary Figures**


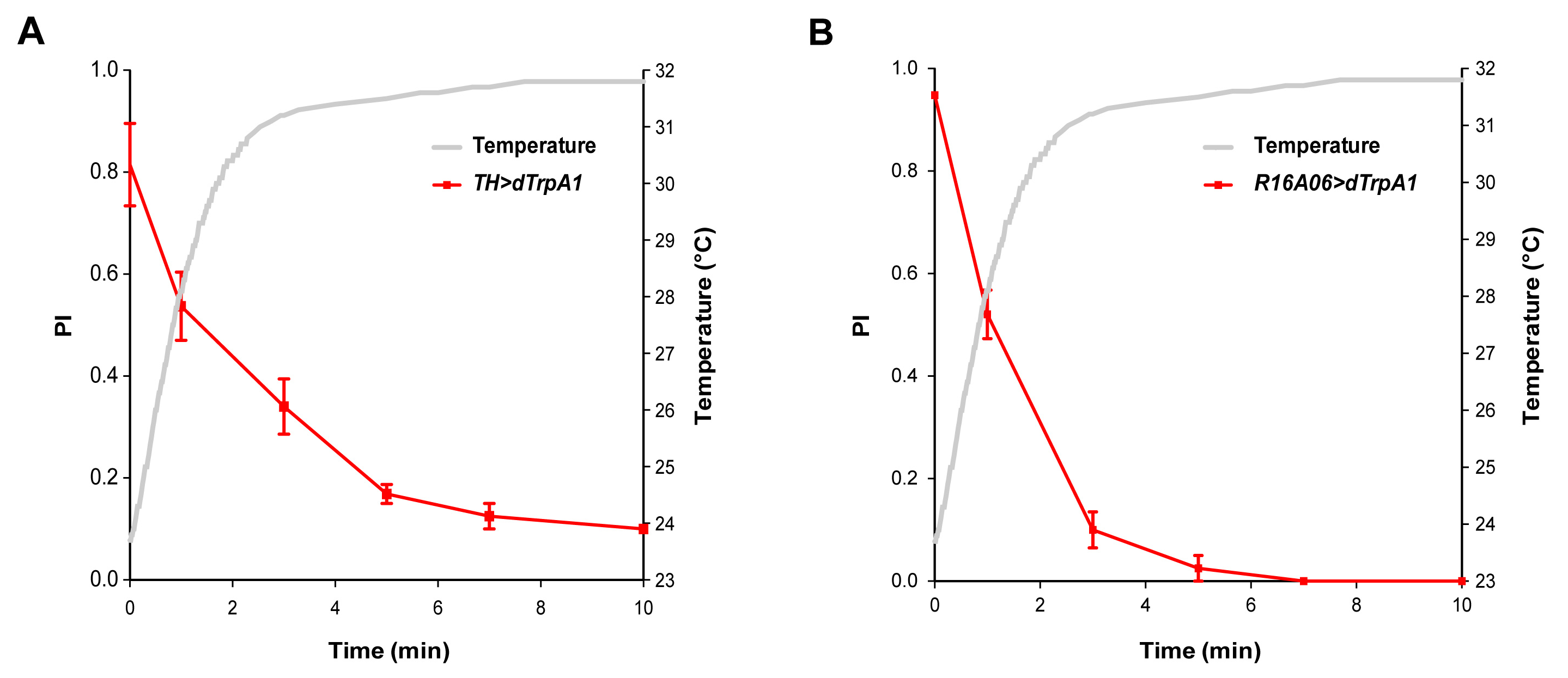


**FIGURE S1 ⏐** Time course of SING behavior modulation during thermogenetic neuronal activation. **(A)** Effect of *TH-Gal4*-targeted DANs. The SING performance of *TH>dTrpA1* flies was determined after various times of incubation at 32°C (red curve). The grey curve shows the kinetics of the temperature increase within the column measured with a thermometer during a 10-min incubation period. A decrease in SING PI could be observed at 1 min compared to the control (time 0) when the temperature of the column reached about 28°C. SING performance did not decrease significantly and stayed stable after 5 min of DAN thermoactivation. **(B)** Effect of MB γ neurons. *R16A06*>*dTrpA1* flies in which γ lobe KCs were selectively thermoactivated showed a more rapid decrease in SING that was ~90% inhibited in 3 min.

**
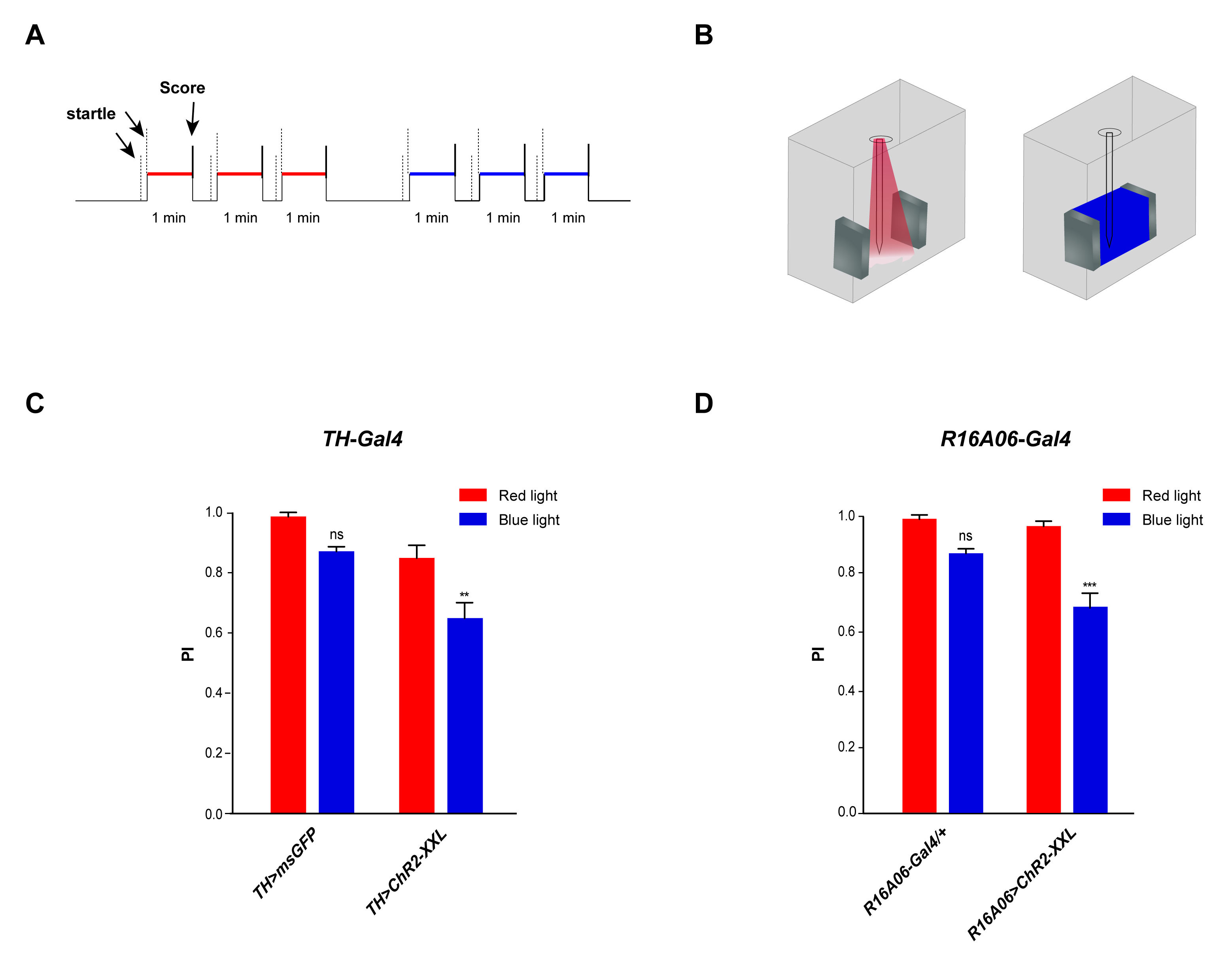
**

**FIGURE S2 ⏐** SING modulation by optogenetic photostimulation. **(A)** Protocol used to assay startle-induced locomotion during optogenetic photoactivation. Mechanical startle was applied just before the column was introduced in the illuminated box and a second time in the box previous to the start of the test (dotted vertical lines). Flies were counted to score PI after 1 min (black vertical lines) using video monitoring. The test was repeated 3 times in red light (control, red horizontal lines) and 3 times in blue light (blue horizontal lines). **(B)** Schematic representation of the experimental setup showing the illuminated dark box with the column inside. Note that the red light covered 100% of the column length and the blue light only 45%. **(C)** Expression of channelrhodopsin in DANs. Photostimulation of *TH>ChR2-XXL* flies with blue light during the test significantly decreased SING performance by ~22% compared to controls illuminated with red light. No significant effect of blue light was observed with control *TH*>*msGFP* flies. **(D)** Expression of channelrhodopsin in MB γ lobes. Photostimulation of *R16A06>ChR2-XXL* flies with blue light during the test decreased SING performance by ~30% compared to controls illuminated with red light. No effect of blue light was observed with control *R16A06-Gal4/+* flies. These experiments indicate that activation of DANs or γ KCs had signifcant effects on startle-induced locomotion within less than 1 min. **(C,D)** Two-way ANOVA with Bonferroni's multiple comparisons tests (ns: not significant; ***p* < 0.01; ****p* < 0.001).

**Movie Captions**

**MOVIE S1 ⏐** Spontaneous locomotion of *TH>dTrpA1* flies after neuronal thermoactivation. Following prolonged thermoactivation of the DANs (10 min at 32°C), *TH>dTrpA1* flies were very active with apparently normal spontaneous locomotion. Note that each column usually contained 10 flies.

**MOVIE S2 ⏐** SING performance of *TH>dTrpA1* flies after neuronal thermoactivation. Suite of Movie S1. After 10-min thermoactivation, flies were gently tapped down (startle), which stops spontaneous locomotion and normally triggers climbing as a locomotor reaction (see Movie S3). Thermoactivated *TH>dTrpA1* flies were still active and showed no signs of paralysis after startle. However, most of them stayed at the bottom of the column instead of climbing up quickly like control flies. A few started to climb but generally stopped in the middle and sometimes fell. This suggests that DAN thermoactivation inhibited startle-induced locomotion.

**MOVIE S3 ⏐** SING performance of control *TH>dTrpA1* flies maintained at 23°C. In the absence of DAN thermoactivation, most of these flies climbed rapidly to the top of the column similarly to wild-type flies.

**MOVIE S4 ⏐** Spontaneous locomotion of *R16A06>dTrpA1* flies after neuronal thermoactivation. After prolonged thermogenetic activation of MB γ neurons (10 min at 32°C), *R16A06*>*dTrpA1* flies were very active and spontaneously walked rapidly along the column. Note that the two columns shown contain flies of the same genotype.

**MOVIE S5 ⏐** SING performance of *R16A06>dTrpA1* flies after neuronal thermoactivation. Suite of Movie S3. Upon startle which stops spontaneous locomotion, *R16A06*>*dTrpA1* flies were still active but their locomotor reactivity appeared to be inhibited as they did not climb efficiently. 10-min thermoactivation of MB γ neurons therefore fully prevented startle-induced locomotion.

**Table S1. Driver strains used in this study and their brain expression patterns.**

| **Drivers** | | **Expression patterns** | | **References** | |
| --- | --- | --- | --- | --- | --- |
| *TH-Gal4* | | All DAN clusters except a major part of the PAM | | Friggi-Grelin et al., 2003; Claridge-Chang et al., 2009; Mao and Davis, 2009; Aso et al., 2010 | |
| *R58E02-Gal4* | | ~80% of PAM DANs | | Liu et al., 2012a; Pech et al., 2013b | |
| *NP6510-Gal4* | | 15 PAM DANs including MB-MVP1 plus 3 non-DANs in the PAM | | Liu et al., 2006; Tanaka et al., 2008; Aso et al. 2010; Riemensperger et al., 2013 | |
| *NP5272-Gal4* | | 3 PAM DANs (MB-M3) that are also labelled by TH-Gal4 | | Tanaka et al., 2008; Aso et al., 2010; Aso et al., 2012 | |
| *Mz840-Gal4* | | PPL1 MB-V1 | | Aso et al., 2010; Aso et al., 2012 | |
| *NP2758-Gal4* | | PPL1 MB-MP1 | | Tanaka et al., 2008; Krashes et al., 2009; Aso et al., 2010 | |
| *TH-D’-Gal4* | PAL, PPM2, PPM3 and PPL1 | | Liu et al., 2012b | |  |
| *TH-C’-Gal4* | | PAL, PPM2 and PPL2ab | | Liu et al., 2012b; Pathak et al., 2015 | |
| *TH-C1-Gal4* | | PAM, PAL, PPM2 and PPL2ab | | Liu et al., 2012b, Kayser et al., 2014 | |
| *TH-LexA* | | Similar to *TH-Gal4* | | Berry et al., 2015 | |
| *238Y-Gal4* | | All MB lobes | | Armstrong et al., 1998; Aso et al., 2009 | |
| *c305a-Gal4* | | MB α'β' lobes and broad expression out of the MB | | Krashes et al., 2007; Aso et al., 2009; Chen et al., 2012; Riemensperger et al., 2013 | |
| *G0050-Gal4* | | MB α'β' lobes | | Lin et al., 2007; Pech et al., 2013b | |
| *4-59-Gal4* | | MB α'β' lobes | | Kaun et al., 2011 | |
| *R35B12-Gal4* | | MB α'β' lobes | | Jenett et al., 2012; Lin et al., 2014; Cohn et al., 2015 | |
| *H24-Gal4* | | MB γ lobes | | Martin et al., 1998; Zars et al., 2000; Aso et al., 2009; Christiansen et al., 2011 | |
| *R16A06-Gal4* | | MB γ lobes | | Jenett et al., 2012; Issman-Zecharya and Schuldiner, 2014; Cohn et al., 2015 | |
| *mb247-Gal4* | | MB αβ and γ lobes | | Zars et al., 2000; McGuire et al., 2001; Aso et al., 2009 | |
| *NP3212-Gal4* | | MBON-M4/M6 | | Tanaka et al., 2008; Bouzaiane et al., 2015 | |
| *R27G01-Gal4* | | MBON-M4/M6 | | Jenett et al., 2012; Bouzaiane et al., 2015 | |
| *R14C08-LexA* | | MBON-M4/M6 | | Jenett et al., 2012; Lewis et al., 2015 | |
| *NP2492-Gal4* | | MBON-V2 | | Tanaka et al., 2008; Séjourné et al., 2011 | |
| *R71D08-Gal4* | | MBON-V2 | | Jenett et al., 2012; Séjourné et al., 2011 | |
| *c41-Gal4* | | EB neurons | | Sakai and Kitamoto, 2006 | |
| *c105-Gal4* | | EB ring neurons R1 | | Renn et al., 1999; Baker et al., 2007; Martín-Peña et al., 2014 | |
| *EB1-Gal4* | | EB ring neurons R2 | | Young and Armstrong, 2010; Thran et al., 2013 | |
| *c232-Gal4* | | EB ring neurons R3/R4d | | Renn et al., 1999; Young and Armstrong, 2010 | |

**Supplementary references**

Armstrong, J. D., de Belle, J. S., Wang, Z., and Kaiser, K. (1998). Metamorphosis of the mushroom bodies; large-scale rearrangements of the neural substrates for associative learning and memory in *Drosophila*. *Learn. Mem.* 5, 102–114.

Aso, Y., Grübel, K., Busch, S., Friedrich, A. B., Siwanowicz, I., and Tanimoto, H. (2009). The mushroom body of adult *Drosophila* characterized by GAL4 drivers. *J. Neurogenet.* 23, 156–172. doi:10.1080/01677060802471718.

Baker, D. A., Beckingham, K. M., and Armstrong, J. D. (2007). Functional dissection of the neural substrates for gravitaxic maze behavior in *Drosophila* melanogaster. *J. Comp. Neurol.* 501, 756–764. doi:10.1002/cne.21257.

Christiansen, F., Zube, C., Andlauer, T. F. M., Wichmann, C., Fouquet, W., Owald, D., et al. (2011). Presynapses in Kenyon cell dendrites in the mushroom body calyx of *Drosophila*. *J. Neurosci.* 31, 9696–9707. doi:10.1523/JNEUROSCI.6542-10.2011.

Cohn, R., Morantte, I., and Ruta, V. (2015). Coordinated and compartmentalized neuromodulation shapes sensory processing in *Drosophila*. *Cell* 163, 1742–1755. doi:10.1016/j.cell.2015.11.019.

Issman-Zecharya, N., and Schuldiner, O. (2014). The PI3K class III complex promotes axon pruning by downregulating a Ptc-derived signal via endosome-lysosomal degradation. *Dev. Cell* 31, 461–473. doi:10.1016/j.devcel.2014.10.013.

Jenett, A., Rubin, G. M., Ngo, T.-T. B., Shepherd, D., Murphy, C., Dionne, H., et al. (2012). A GAL4-driver line resource for *Drosophila* neurobiology. *Cell Rep* 2, 991–1001. doi:10.1016/j.celrep.2012.09.011.

Kaun, K. R., Azanchi, R., Maung, Z., Hirsh, J., and Heberlein, U. (2011). A *Drosophila* model for alcohol reward. *Nat Neurosci* 14, 612–619. doi:10.1038/nn.2805.

Kayser, M. S., Yue, Z., and Sehgal, A. (2014). A critical period of sleep for development of courtship circuitry and behavior in *Drosophila.* *Science* 344, 269–274.

Lin, A. C., Bygrave, A. M., de Calignon, A., Lee, T., and Miesenböck, G. (2014). Sparse, decorrelated odor coding in the mushroom body enhances learned odor discrimination. *Nat. Neurosci.* 17, 559–568. doi:10.1038/nn.3660.

Liu, G., Seiler, H., Wen, A., Zars, T., Ito, K., Wolf, R., et al. (2006). Distinct memory traces for two visual features in the *Drosophila* brain. *Nature* 439, 551–556. doi:10.1038/nature04381.

Martín-Peña, A., Acebes, A., Rodríguez, J.-R., Chevalier, V., Casas-Tinto, S., Triphan, T., et al. (2014). Cell types and coincident synapses in the ellipsoid body of *Drosophila*. *Eur J Neurosci* 39, 1586–1601. doi:10.1111/ejn.12537.

McGuire, S. E., Le, P. T., and Davis, R. L. (2001). The role of *Drosophila* mushroom body signaling in olfactory memory. *Science* 293, 1330–1333. doi:10.1126/science.1062622.

Pathak, T., Agrawal, T., Richhariya, S., Sadaf, S., and Hasan, G. (2015). Store-operated calcium entry through orai is required for transcriptional maturation of the flight circuit in *Drosophila*. *J. Neurosci.* 35, 13784–13799. doi:10.1523/JNEUROSCI.1680-15.2015.

Renn, S. C., Armstrong, J. D., Yang, M., Wang, Z., An, X., Kaiser, K., et al. (1999). Genetic analysis of the *Drosophila* ellipsoid body neuropil: organization and development of the central complex. *J. Neurobiol.* 41, 189–207.

Sakai, T., and Kitamoto, T. (2006). Differential roles of two major brain structures, mushroom bodies and central complex, for *Drosophila* male courtship behavior. *J. Neurobiol.* 66, 821–834. doi:10.1002/neu.20262.

Thran, J., Poeck, B., and Strauss, R. (2013). Serum response factor-mediated gene regulation in a *Drosophila* visual working memory. *Curr. Biol.* 23, 1756–1763. doi:10.1016/j.cub.2013.07.034.

Young, J. m., and Armstrong, J. d. (2010). Structure of the adult central complex in *Drosophila*: Organization of distinct neuronal subsets. *J. Comp. Neurol.* 518, 1500–1524. doi:10.1002/cne.22284.

Zars, T., Fischer, M., Schulz, R., and Heisenberg, M. (2000). Localization of a short-term memory in *Drosophila*. *Science* 288, 672–675.
